# The effect of random shocks on reciprocal behavior in dynamic principal-agent settings

**DOI:** 10.1007/s10683-022-09771-w

**Published:** 2022-10-05

**Authors:** Rudolf Kerschbamer, Regine Oexl

**Affiliations:** Department of Economics, University of Innsbruck, Innsbruck, Austria

**Keywords:** Gift exchange, Principal agent model, Incomplete contracts, Random shocks, Reciprocity, Laboratory experiments, Long-term contracts, C72, C91, D81

## Abstract

Previous work has shown that unobservable random shocks on output have a detrimental effect on efficiency in short-term (‘static’) employment relationships. Given the prevalence of long-term (‘dynamic’) relationships in firms, we investigate whether the impact of shocks is similarly pronounced in gift-exchange relationships where the same principal-agent pair interacts repeatedly. In dynamic relationships, shocks have a significantly less pronounced negative effect on efficiency than in static relationships. In an attempt to identify the drivers for our results we find that the combination of a repeated-game effect (current misbehavior can be punished in future periods) and a noise-canceling effect (part of the noise cancels out in the long run) is required to avoid the detrimental effects of unobservable random shocks on efficiency.

**Supplementary Information:**

The online version contains supplementary material available at 10.1007/s10683-022-09771-w.

## Introduction

A large literature studies how mutual gift-exchange based on reciprocity can improve the relationship between parties with conflicting interests, such as employers (henceforth principals) and workers (henceforth agents). Starting with the seminal study by Fehr et al. (1993), many experimental papers find a positive association between the wage the principal offers and the effort the agent exerts in response in a two-stage gift-exchange game (for reviews, see Gächter & Falk, 2002; Fehr et al., 2009; Charness & Kuhn, 2011). This link is even more pronounced when the principal additionally can reward or punish the agent for his effort in a three-stage gift-exchange game (Fehr et al., 1997, 2007, 2007). An important aspect of the gift-exchange mechanism in both cases is that it is effective even in short-term relationships where contract enforcement based on reputational concerns is not available.

In a thought-provoking paper, Rubin and Sheremeta (2016) show that mutual gift-exchange may lose its effectiveness when unobservable random shocks obscure the relation between the agent’s effort and output. Specifically, the authors conduct a three-stage gift-exchange experiment similar to that in Fehr et al. (1997), except that the principal cannot directly observe the agent’s action, but only a noisy signal.^1^ Gift-exchange and welfare are significantly depressed in this setting compared to the same situation without random shocks. Davis et al. (2017) confirm this result in a replication study. This finding is concerning, since in many real-world relationships the outcome from exchange is subject to random shocks and cannot cleanly be attributed to kind or unkind acts. However, while short-term (static) interactions with random shocks are certainly relevant, many real world interactions last longer than one period. An interesting question therefore is whether the adverse effects of unobservable random shocks are contained in long-term (dynamic) gift-exchange interactions. The present paper addresses this question in a series of lab experiments.

In our experiments, we follow Rubin and Sheremeta (2016) and Davis et al. (2017) in employing a three-stage gift-exchange game, in which both sides of the market—the principal and the agent—can respond reciprocally to previous actions. We do so for several reasons. First, the three-stage nature of the game does not change the standard prediction (based on the assumption that it is common knowledge that all players are exclusively interested in their own material payoffs) for the two stages in the standard gift-exchange game: a purely self-interested principal would not implement a (costly) bonus or fine in the third stage; anticipating this, the self-interested agent would have no incentive to provide effort above the minimum in the second stage; and the principal would in turn have no incentive to offer a wage above the minimum in the first stage. Second, we think that the three-stage game better reflects real-world employment interactions: In some real world employment relationships based on reciprocity there is an explicit third stage, as voluntary bonus payments are an important component of the overall compensation package of the employee. In other relationships, there is implicitly a third stage, as for agents motivated by social concerns, a friendly (or unfriendly) word when leaving the contract may represent a ‘bonus’ (or ‘fine’). Third and most importantly, it has been shown that reciprocal behavior is stronger in a three-stage game than in a two-stage game (Fehr et al., 1997, 2007; Ernst Fehr, 2004). This makes the results of Rubin and Sheremeta (2016) and Davis et al. (2017) even more remarkable: Even in those static relationships where reciprocity has been shown to be a powerful means to improve efficiency, unobservable random shocks have a detrimental effect on efficiency. This makes the three-stage game particularly suitable to study the impact of extending the relationship length to see whether the adverse effects of unobservable random shocks are contained in long-term interactions.

Neither Rubin and Sheremeta (2016) nor Davis et al. (2017) directly address the question why gift-exchange is depressed—i.e., why wage and effort are lower—in the presence of unobservable random shocks. One possibility is that agents no longer trust in reciprocal acts by principals, when the latter cannot disentangle the agent’s effort from good or bad luck. This might diminish the agent’s effort motivation, which in turn might lead the (anticipating) principal to offer a less generous wage arrangement in the first place. If this explanation were true, then gift-exchange might be restored in a dynamic relationship, since part of the noise cancels out in the long run.^2^ We call this the *noise-canceling effect*. The noise-canceling effect potentially has two components, a passive one and an active one. The passive component is present even when the agent keeps the effort constant over time: By observing several outputs the principal gets more information about the agent’s behavior over time. We call this part of the noise-canceling effect the *learning component*. In addition to the learning component there might also be an active component of the noise-canceling effect: The agent might react to a negative (positive) shock in the previous period by exerting more (less) effort in the current period—thereby protecting (at least in part) the principal from the shock. We call this part of the noise-canceling effect the *insurance component*.^3^ Gift exchange might also work better in a dynamic relationship because current misbehavior can be punished in future periods. In the limit (when the relationship lasts infinitely long) this allows for some kind of forcing contracts à la folk theorem, where both partners do not want to risk the benefits from future gift-exchange by committing adverse acts in the current period.^4^ We call this the *repeated-game effect*. While the repeated-game effect is predicted to be present in dynamic employment relationships independently of whether they are plagued by unobservable random shocks or not, the noise-canceling effect is by definition present only in dynamic interactions plagued by unobservable random shocks.

To separate the noise-canceling effect from the repeated-game effect, our main experiments are based on a 2 × 2 design. In one dimension we vary whether unobservable random shocks are absent (in this case the agent’s effort can be perfectly inferred from the output) or present (in this case the output is only a noisy signal of effort), in the other dimension we vary whether the interaction is static (each principal-agent pair plays the gift-exchange game only once) or dynamic (a principal-agent pair interacts over several periods). We isolate the repeated-game effect by comparing behavior and overall efficiency in the dynamic principal-agent relation without random shocks to its static counterpart. To receive aggregate information about the importance of the noise-canceling effect, we use a difference-in-difference approach: We compare the difference in behavior and efficiency between the dynamic principal-agent relation with random shocks and its static counterpart (where both the repeated-game effect and the noise-canceling effect might play a role) to the difference between the dynamic principal-agent relation without random shocks and its static counterpart (where arguably only the repeated-game effect is at work).

We also search for direct evidence for the insurance component of the noise-canceling effect and for the repeated-game effect in our data. The insurance component implies that current effort is negatively related to the size of the shock in the previous period—a part of the shock is in effect absorbed by the agent. By contrast, the repeated-game effect predicts that current effort is positively related to previous adjustment and that the current wage is positively related to previous output.^5^

Our results are as follows: While we find some direct evidence for the presence of a repeated-game effect in our treatments without random shocks, the effect seems to be insufficient to make the dynamic relationship more efficient than the static one. Indeed, in the absence of shocks, extending the relationship length does neither increase the average wage, nor the average effort, nor the average adjustment. As a consequence, overall efficiency is also not significantly different between the two treatments without random shocks. This result is probably due to the fact that the three-stage gift-exchange game already leaves sufficient possibilities to reward and punish behavior within a single round.

By contrast, in the presence of unobservable random shocks the dynamic interaction is significantly more efficient than the static one. Comparing treatments without random shocks to those with shocks, we find that unobservable random shocks have a pronounced negative effect on efficiency in the static interaction (the results reported in Rubin & Sheremeta, 2016; Davis et al., 2017), but efficiency is roughly the same across the two dynamic interactions. These results together suggest that the noise-canceling effect is mainly responsible for the result that in dynamic relationships, shocks have a significantly less pronounced negative effect on efficiency than in static relationships.

To address the question whether noise-canceling alone is sufficient to eliminate the negative effect, we run two additional treatments—the ‘no-repeated-game-effect treatments’. In both, the interaction is dynamic, and, again, we vary whether there are unobservable random shocks on effort or not. In contrast to the dynamic treatments, in these two additional treatments, a principal-agent pair interacts under the *same* contract over the whole duration of the relationship. That is, the principal offers a wage and states a desired effort at the beginning of the relationship, and these values are valid for each of the periods the principal and the agent interact. The agent then chooses an effort in every period, and in the treatment with shocks, also the shock is realized each period. In this setup, the two components of the noise-canceling effect—that is, the learning component and the insurance component—potentially are still active, while the repeated-game effect is turned off. While we find some direct evidence for the presence of the noise-canceling effect in the data of the no-repeated-game-effect treatment with random shocks, the effect seems to be insufficient to neutralize the negative impact of random shocks on efficiency. Indeed, in the no-repeated-game-effect condition, the presence of unobservable shocks has a similarly pronounced negative effect on efficiency as in the static interaction.

Taken together, our results indicate that neither the repeated-game effect alone nor the noise-canceling effect alone is sufficient to alleviate the detrimental effects of unobservable random shocks on efficiency. What is needed to create a setting where unobserved random shocks do not impact reciprocal behavior substantially is an environment in which both the repeated-game effect and the noise-canceling effect can be active.

Turning to related literature, our results may help to understand recent findings from field experiments: Gneezy and List (2006) run a field experiment aimed at increasing worker effort in two quite distinct tasks: data entry for a university library and door-to-door fundraising for a research center. In both settings the authors offer individuals either the wage as announced (no-gift condition) or a higher wage (gift condition). The authors find for both tasks that worker effort in the first few hours on the job is considerably higher in the gift condition than in the no-gift condition. However, the effect fades out after a few hours, and for later hours no difference in outcomes is observed. In line with this, de Ree et al. (2018) find in a sample of teachers in India that while an unconditional salary increase does improve teacher satisfaction and other measures in the short run, it does not impact student performance in the long run. While these papers do not explicitly discuss the presence of random shocks in their environments, some elements of randomness are clearly present.^6^ Our results suggest that the fading out of the effect found in this literature might be due to the missing repeated-game effect: regularly adjusting the wage based on the observed performance might restore gift-exchange.

Turning to related laboratory experiments, the papers closest to ours are Rubin and Sheremeta (2016) and Davis et al. (2017). These papers study the impact of unobservable random shocks on behavior in static gift-exchange games but do not consider dynamic interactions. Dynamic gift-exchange games have been investigated by Falk et al. (1999), Gächter and Falk (2002), and Brown et al. (2004). In contrast to our experimental design these papers investigate only gift-exchange games without random shocks. Another difference is that the basic game implemented in those papers is a two-stage interaction where the principal offers a wage in the first stage and the agent decides about her effort in the second stage. By contrast our basic game has a third stage in which the principal can reward or punish the agent after seeing her effort choice. This latter difference might explain why we do not find a repeated-game effect in our treatments without random shocks while the mentioned papers find that extending the relationship length fosters reciprocal behavior and leads to more efficient outcomes. To the best of our knowledge there is no experimental literature on the effects of unobservable random shocks in dynamic gift-exchange games.

The rest of the paper is organized as follows. Section 2 describes the experimental design, the four main treatments and the procedures. Section 3 reports the results. In Sect. 4, we introduce two additional treatments and investigate the impact of removing the repeated-game effect on gift-exchange relationships plagued by random shocks. Section 5 concludes.

## Experimental design, treatments and procedures

*The baseline game* Our baseline game of a static interaction without shocks is identical to the baseline game in Davis et al. (2017): The game has three stages. In stage one, the principal (she) offers a contract $(w,e^*)$, specifying a wage $w\in \{1,2,\ldots ,100\}$ and an (unenforceable) desired effort $e^*\in \{0,1,\ldots ,14\}$ that she would like the agent to undertake. In stage two, the agent (he) observes the contract chosen by the principal and decides about the effort level $e\in \{0,1,\ldots ,14\}$. The cost of effort—$c_e(e) = e^{2}/2$, rounded to the next highest integer—is common knowledge among the players, as are all other details. In stage three, the principal observes the outcome *y* and chooses an adjustment level $a \in \{-50, -40,\ldots , 0,\ldots ,40,50\}$, where positive values are bonuses to the agent and negative values are fines. Adjustments are costly for the principal, with an adjustment cost of $c_a(a) = \frac{|a|}{10}$.

**Table 1:** Overview main treatments

	No-shock	Shock
Static	S$_{\text {no-shock}}$	S$_{\text {shock}}$
Dynamic	D$_{\text {no-shock}}$	D$_{\text {shock}}$

In *static* interactions a principal-agent pair interacts only once (i.e., in a single period); in *dynamic* interactions a principal-agent pair remains intact for five periods; *no-shock* refers to an interaction where the effort translates directly into output; *shock* refers to an interaction where output is composed by the sum of effort and shock

*The roles and the periods* Individuals play over 10 periods; the player roles (agent or principal) are assigned before the first period and stay constant for the remaining periods.

*The four main treatments* Our main design comprises four treatments. We vary two dimensions of the principal-agent relationship, as summarized in Table 1. The first dimension is whether a shock occurs (‘shock’) or not (‘no-shock’); the second dimension is whether the relationship lasts for one period (‘S’, for static relationship), or for five periods (‘D’, for dynamic relationship). In the following we refer to a series of five periods as ‘one block’.

The variation on the shock refers to how effort translates into outcome: In the ‘no-shock’ treatments, the outcome *y* corresponds to the effort *e*; in the ‘shock’ treatments, *y* is the sum of *e* and an uniformly distributed random integer component $\epsilon _i \in \{-2, -1, 0, 1,2\}$. The variation on the duration of the relationship refers to the length of the interaction within the same principal-agent pair. In the ‘S’ treatments, subjects form groups of eight (four agents, four principals) and are randomly rematched within their group with a partner of the other role at *the end of each period*. In the ‘D’ treatments, subjects form groups of four (two agents, two principals) and are rematched within their group at *the end of a block*; that is, a principal-agent pair remains intact for five periods.

*The payoffs* In all treatments one randomly selected period is chosen for payment; in this period, the payoff function is $\pi ^P = 10y - w - c_a(a)$ for the principal and $\pi ^A = w - c_e(e) + a$ for the agent.

*Information provided* In all treatments, the agents receive information about the wage and the desired effort before making their effort decision and the principals receive information about the wage, the desired effort and the output (which corresponds to the effort in the no-shock treatments and to effort plus shock in the shock treatments) before making their adjustment decision. In addition, all participants receive the following information at the end of each period: the wage; the desired effort; the output; the adjustment; as well as the individual earnings for that period. After each period, participants have the opportunity to record this information in a personal recording sheet.

*Procedures* The experiment was programmed in z-Tree (Fischbacher, 2007) and participants were recruited via hroot (Bock et al., 2014). Sessions were run at the Innsbruck EconLab and lasted on average around 70 minutes. Average earnings per participant were €13.83. We ran three sessions à 24 subjects per treatment; with three matching groups of eight in the S sessions (respectively, six matching groups of four in the D sessions) this results in 9 (respectively 18) independent observations per treatment, when using the average within a single matching group over all periods as one independent observation.^7^

*Variables of interest* Our main variables of interest are the effort and the adjustment, since these two variables reveal reciprocity towards kind or unkind behavior in the previous stage. In addition, these are the productive actions, in the sense that they directly affect efficiency. Furthermore, we report the wage, since both effort as well as adjustment may be influenced by first stage behavior. Finally, we report total welfare, defined as the sum of the payoffs, as this is our measure of efficiency.

## Results

### The impact of extending the relationship length

**Table 2:** Averages and Mann-Whitney U-tests regarding decision variables and welfare

		Wage	Effort	Adjustment	Welfare
*Averages*					
S	No-shock	35.19	5.81	6.96	39.96
		(3.07)	(0.49)	(2.97)	(5.00)
	Shock	29.21	4.20	− 1.28	23.81
		(3.00)	(0.43)	(2.20)	(3.70)
D	No-shock	35.61	5.46	4.72	33.75
		(2.56)	(0.31)	(1.96)	(2.44)
	Shock	35.48	5.56	8.11	39.89
		(2.38)	(0.33)	(1.92)	(3.18)
*P-values of Mann-Whitney U-tests comparing averages*					
(1) S$_{\text {no-shock}}$ vs. D$_{\text {no-shock}}$		0.88	0.54	0.43	0.33
(2) S$_{\text {no-shock}}$ vs. S$_{\text {shock}}$		0.20	0.02	0.06	0.05
(3) D$_{\text {no-shock}}$ vs. D$_{\text {shock}}$		0.72	0.76	0.22	0.19

Standard errors in parenthesis are based on 9 independent observations in the S treatments and on 18 indep. observations in the D treatments; these are also the number of observations used for the Mann-Whitney U-tests

**Fig. 1:**
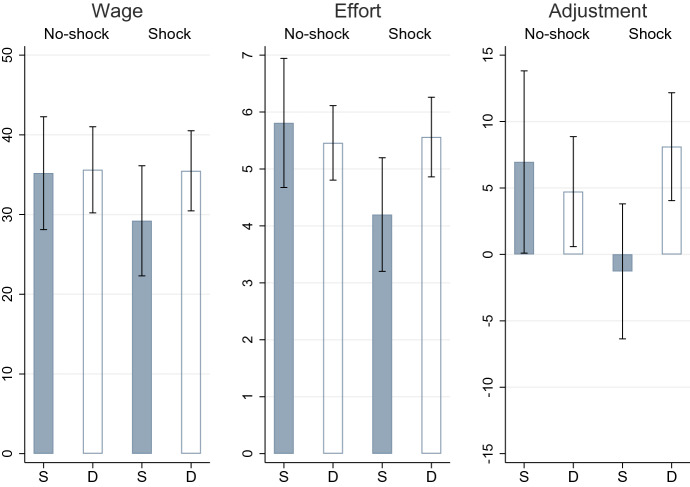
Wage, effort, and adjustment, with error bars representing the 95% conf. intervals

We investigate the effect of extending the relationship length in an environment without shocks by comparing the S$_{\text {no-shock}}$ to the D$_{\text {no-shock}}$ data. Averages of the main variables of interest are summarized in Table 2 and Fig. 1. The lower part of Table 2 reports the *p*-values of Mann-Whitney U-tests (MWU-tests) comparing averages. Average wage, average effort and average adjustment do not differ significantly between the two treatments (see row (1) of the bottom part of Table 2). As a consequence, average welfare does not differ between the treatments, either. We record this in Result 1:

#### Result 1

In an environment without unobservable random shocks, extending the relationship length has no significant effect on average wage, average effort, and average adjustment. As a consequence, total welfare does not differ significantly between the static and the dynamic relationship without random shocks.

Result 1 is in contrast to findings in the previous literature (see, for instance, Falk et al., 1999; Gächter & Falk, 2002; Brown et al., 2004). This is probably due to the fact that the previous literature uses the *two-stage* gift-exchange game as the basic game, while our basic game has three stages. Adding a third stage allows the principal to punish or reward the agent’s effort choice within a given round, and this has been shown to have a pronounced effect on efficiency see, for instance Fehr et al., 1997, 2004, 2007). This might explain why extending the relationship length does not have a significant additional effect.

In online Appendix A.1 we investigate the determinants of effort and adjustment, by estimating different panel models where standard errors are clustered at the matching group level and calculated using a bootstrap method (Cameron et al., 2008). In line with previous literature (Fehr et al., 1997; Rubin & Sheremeta, 2016), we regress the adjustment in the third stage on the difference between effort and desired effort (plus some control variables). As expected, the difference between effort and desired effort has a significant positive impact on adjustment in both treatments. In the investigation of the determinants of the agent’s effort choice in stage two, we regress effort on wage and desired effort and on some control variables. As expected, average effort is significantly higher when the wage is higher, and desired effort has a significant positive impact on effort in both treatments. See online Appendix A.1 for a more thorough discussion of the results.

Next we search for direct evidence for the presence of a repeated-game effect. The repeated-game effect would predict that in a dynamic relationship, the agent’s effort in the current period is positively related to the adjustment in the previous period: The agent responds to a generous (low) adjustment in the previous period by exerting more (less) effort in the current period. We would not expect such a relationship in the static treatment, since the agent is paired with a new partner in each period. The repeated-game effect would also predict that the wage in the current period is positively related to the output in the previous period: The principal responds to the high output in the previous period by offering a more (less) generous wage in the current period. Again, no such relationship is expected for the static treatment. We display those predictions in columns (1) and (5) of Table 3 and in columns (2) and (6) we summarize our main results regarding those predictions.

**Table 3:** Repeated-game effect: predictions and results

	(1)	(2)	(3)	(4)	(5)	(6)	(7)	(8)
	S$_{\text {no-shock}}$		S$_{\text {shock}}$		D$_{\text {no-shock}}$		D$_{\text {shock}}$	
	Prediction	Evidence	Prediction	Evidence	Prediction	Evidence	Prediction	Evidence
Effort & adjustment$$_{\text {t-1}}$$	No correl.	0.01$$^{*}$$ (0.01)	No correl.	0.01 (0.01)	**+**	0.02$$^{***}$$ (0.01)	**+**	0.03$$^{***}$$ (0.01)
		*T*4 (1)		*T*7 (1)		*T*4 (2)		*T*7 (2)
Wage & output$$_{\text {t-1}}$$	No correl.	0.34 (0.30)	No correl.	0.50 (0.37)	**+**	2.91$$^{***}$$ (0.41)	**+**	2.49$$^{***}$$ (0.26)
		*T*4 (4)		*T*7 (3)		*T*4 (5)		*T*7 (4)

We display a ‘+’ if we expect a positive correlation. As evidence we display the coefficients of the regressions (with standard errors in parenthesis). In italics, we report the table (‘T’) and the corresponding column in parenthesis in which the result is reported. The significance levels are: * p<0.10, ** p < 0.05, and *** p < 0.01

We first present our results regarding the repeated-game effect in the effort provision. In the dynamic no-shock treatment, there is a significant positive correlation between current effort and previous adjustment—see column (2) of Table 4. However, the effect is not large (the coefficient is of size 0.02 and explains around 6 percentage points of the variance unexplained by other factors), and, contrary to our prediction, it is also present (and of similar size) in the static no-shock treatment (although only significant at the 10% level—see columns (1) and (3) of Table 4). This latter finding suggests that the positive correlation between current effort and previous adjustment in the no-shock treatments is not due to the repeated-game effect but rather due to experience: when the effort in the previous period was rewarded by a high adjustment in the previous period, agents are willing to provide high effort also in the current period.

Columns (4) and (5) of Table 4 provide more convincing evidence for the existence of a repeated-game effect. We regress wage on past-period output and past-period adjustment, as well as on some control variables. In the dynamic treatment, the current wage is significantly higher when the previous output was higher, while there is no statistically significant relationship between current wage and past-period output in the static treatment.

**Table 4:** Panel model of effort and wage, controlling for past-period behavior, no-shock treatments

Dep. variable	(1)	(2)	(3)	(4)	(5)
	S	D	S and D	S	D
	Effort	Effort	Effort	Wage	Wage
Adjustment$$_{\text {t-1}}$$	0.01$$^{*}$$	0.02$$^{***}$$	0.02$$^{***}$$	0.02	− 0.01
	(0.01)	(0.01)	(0.01)	(0.04)	(0.06)
Wage	0.08$$^{***}$$	0.08$$^{***}$$	0.08$$^{***}$$		
	(0.01)	(0.01)	(0.01)		
Desired effort	0.09	0.18$$^{***}$$	0.14$$^{***}$$		
	(0.07)	(0.05)	(0.04)		
T$_D$			− 0.28		
			(0.39)		
Adjustment$_{\text {t-1}}$ × T$_D$			0.00		
			(0.01)		
Output$$_{\text {t-1}}$$				0.34	2.91$$^{***}$$
				(0.30)	(0.41)
Risk aversion	− 0.00	− 0.00	− 0.00	0.06$^{*}$	− 0.00
	(0.01)	(0.01)	(0.01)	(0.03)	(0.04)
Inv. period	− 1.52	− 4.22$$^{***}$$	− 2.95$$^{***}$$	33.42$$^{***}$$	5.22
	(1.19)	(1.37)	(0.92)	(8.39)	(9.33)
Constant	2.76$$^{***}$$	2.08$$^{***}$$	2.46$$^{***}$$	22.33$$^{***}$$	18.82$$^{***}$$
	(0.84)	(0.56)	(0.53)	(3.51)	(3.59)
Observations	324	324	648	324	324

Standard errors in parentheses are clustered on the group level and calculated via bootstrap. Inv. period runs from 1 to 1/10. Risk aversion runs from 1 to 100, with higher numbers indicating less risk aversion. Wage runs from 0 to 100. Effort and desired effort run from 0 to 14. Adjustment runs from -50 to 50. T$$_D$$ is a dummy variable equal to one if the treatment is dynamic and zero otherwise. Output$$_{\text {t-1}}$$ is the output of the previous period. In the no-shock treatments, output corresponds to effort. The significance levels are: $$^{*}$$ $$p~<~0.10$$, $$^{**}$$ $$p~<~0.05$$, $$^{***}$$ $$p~<~0.01$$

#### Result 2

In the no-shock treatments, we find direct evidence for the presence of a repeated-game effect in the wage determination in the dynamic but not in the static interaction. There is also some evidence for a repeated-game effect in effort provision, but the effect is weak and (contrary to the prediction) also present in the static interaction.

Summarizing the results of this section, we conclude that there is some direct evidence for the presence of a repeated-game effect in the data of the no-shock treatments (as summarized in Result 2). However, the effect seems to be insufficient to make the dynamic relationship more efficient than the static one (Result 1).Ñ

### The impact of shocks on behavior

As shown by Rubin and Sheremeta (2016) and Davis et al. (2017), in static interactions the presence of unobservable random shocks has a pronounced negative impact on efficiency. In our experimental design this result is reflected in the comparison between the S$_{\text {no-shock}}$ and the S$_{\text {shock}}$ treatment: The presence of shocks reduces the average wage by 16.99%, the average effort by 27.71%, the average adjustment by 118.39%, and average welfare by 40.41%. While the decrease in effort, adjustment and welfare is significant, the decrease in wage is not (see row (2) of the bottom part of Table 2).^8^

Turning to the D treatments, we find that the presence of unobservable random shocks does not have a significant impact on wage, effort, adjustment, or welfare (see row (3) of the bottom part of Table 2).

We next compare the difference between the D$_{\text {shock}}$ and the S$_{\text {shock}}$ treatment to the difference between the D$_{\text {no-shock}}$ and the S$_{\text {no-shock}}$ treatment. From the above results we expect that extending the relationship length has a significant positive effect on our main variables of interest in the treatments with random shocks. Instead, from Result 1 we know that extending the relationship length has no significant effects on our variables of interest in the treatments without random shocks. As a result, we would expect that compared to the treatments without random shocks, extending the relationship length has a more positive impact on wage, effort, adjustment, and welfare in the treatments with unobservable random shocks. This is indeed the case—with one qualification: While the diff-in-diff OLS regression shows significant differences in effort, adjustment, and welfare (see the differences reported in rows (1) and (2) of Table 5, and the regression results reported in Table 17 in online Appendix B), the difference in wage is not significant.

**Table 5:** Differences between the treatments regarding decision variables and welfare

	Wage	Effort	Adjustment	Welfare
(1) D$_{\text {shock}}$ − S$_{\text {shock}}$	6.27	1.36	9.39	16.08
(2) D$_{\text {no-shock}}$ − S$_{\text {no-shock}}$	0.42	− 0.35	− 2.23	− 6.21
(3) D$_{\text {shock}}$ − D$_{\text {no-shock}}$	− 0.13	0.10	3.39	6.15
(4) S$_{\text {shock}}$ − S$_{\text {no-shock}}$	− 5.98	− 1.61	− 8.23	− 16.16
(5) NRG$_{\text {shock}}$ − NRG$_{\text {no-shock}}$	− 7.50	− 1.49	4.03	1.11

Averages are based on 9 independent observations in the S treatments and on 18 indep. observations in the D and NRG treatments

We summarize our findings regarding the impact of random shocks as follows:

#### Result 3

The presence of unobservable random shocks has a pronounced negative effect on efficiency when the relationship is static but no significant effect on efficiency when the relationship is dynamic. Comparing the effect of extending the relationship length between the environment with random shocks and the environment without random shocks we find a significantly more pronounced positive effect on efficiency in the presence of random shocks than in the absence of random shocks.

**Table 6:** Noise-canceling effect: predictions and results

	(1)	(2)	(3)	(4)	(5)	(6)
	S$_{\text {shock}}$		D$_{\text {shock}}$		NRG$_{\text {shock}}$	
	Prediction	Evidence	Prediction	Evidence	Prediction	Evidence
Effort & Shock$$_{\text {t-1}}$$	No correl.	0.12 (0.09)	–	− 0.21$$^{*}$$ (0.12)	–	− 0.13$$^{**}$$ (0.06)
		*T7 (1)*		*T7 (2)*		*T7 (1)*

We display a ‘–’ if we expect a negative correlation. As evidence we display the coefficients of the regressions (with standard errors in parenthesis). In italics, we report the table (‘T’) and the corresponding column in parenthesis in which the result is reported. The significance levels are: * p<0.10, ** p < 0.05, and *** p < 0.01

In online Appendix A.2 we investigate the determinants of effort and adjustment, by estimating different panel models based on data from both the shock and the no-shock treatment. We regress the adjustment on the difference between output and desired effort, on wage, on a ‘T$_{\text {shock}}$’ dummy, on the respective interaction terms, and on control variables. We find no significant difference in the impact of ‘output - desired effort’ between the shock and the no-shock treatments, and as expected, the difference between output and desired effort positively correlates with the adjustment in all treatments. Furthermore, we regress effort on wage, desired effort, the T$_{\text {shock}}$ dummy, interaction terms of these variables, and control variables. The impact of wage on effort does not differ significantly between the two treatments, and, as expected, the impact of wage is positive in all settings. We discuss further details in online Appendix A.2.

**Table 7:** Panel model of effort and wage, controlling for shock and past-period behavior, shock treatments

Dep. variable	(1)	(2)	(3)	(4)
	S	D	S	D
	Effort	Effort	Wage	Wage
Adjustment$$_{\text {t-1}}$$	0.01	0.03$$^{***}$$	0.02	0.02
	(0.01)	(0.01)	(0.04)	(0.03)
Wage	0.08$$^{***}$$	0.09$$^{***}$$		
	(0.01)	(0.01)		
Desired effort	0.03	0.13$$^{**}$$		
	(0.03)	(0.05)		
Shock$_{\text {t-1}}$	0.12	− 0.21$^{*}$		
	(0.09)	(0.12)		
Output$$_{\text {t-1}}$$			0.50	2.49$$^{***}$$
			(0.37)	(0.26)
Risk aversion	0.02$$^{**}$$	− 0.00	0.09	− 0.11$$^{**}$$
	(0.01)	(0.01)	(0.07)	(0.05)
Inv. period	− 0.33	− 0.19	13.76$$^{***}$$	11.36$$^{*}$$
	(1.46)	(1.08)	(4.58)	(6.64)
Constant	0.37	1.36$$^{**}$$	18.57$$^{***}$$	23.84$$^{***}$$
	(0.67)	(0.65)	(5.33)	(3.65)
Observations	324	324	324	324

Standard errors in parentheses are clustered on the group level and calculated via bootstrap. Inv. period runs from 1 to 1/10. Risk aversion runs from 1 to 100, with higher numbers indicating less risk aversion. Wage runs from 0 to 100. Effort and desired effort run from 0 to 14. Adjustment$$_{\text {t-1}}$$ is the adjustment of the previous period and runs from− 50 to 50. Shock$$_{\text {t-1}}$$ is the shock of the previous period and runs from − 2 to 2. Output$$_{\text {t-1}}$$ is the output of the previous period. In the shock treatments, output corresponds to effort plus the shock. The significance levels are: $$^{*}$$ $$p~<~0.10$$, $$^{**}$$ $$p~<~0.05$$, $$^{***}$$ $$p~<~0.01$$

Next we investigate whether there is direct evidence for the repeated-game effect and for the active part of the noise-canceling effect in dynamic relationships with random shocks. The correlations predicted by the repeated-game effect are as described in Sect. 3.1 and displayed in columns (3) and (7) of Table 3; columns (4) and (8) record the main findings. The active (or ‘insurance’) component of the noise-canceling effect predicts that in dynamic relationships, the agent reacts to a negative (positive) shock in the previous period with higher (lower) effort in the current period. In a static relationship, since the agent is paired with a new partner in each period, there is no reason to expect such ‘smoothing’ behavior. Columns (1) and (3) of Table 6 display these predictions, and columns (2) and (4) record our main findings in this respect.

In columns (1) and (2) of Table 7 we regress the effort on wage, desired effort, the adjustment of the previous period, the size of the shock of the previous period, and some other variables, for each of the two shock treatments separately. The past-period shock correlates negatively with current-period effort in D$_{\text {shock}}$, but not in S$_{\text {shock}}$, which is in line with our hypothesis that there is an insurance component in dynamic but not in static relationships plagued by random shocks. We also see that the past-period adjustment correlates positively with current-period effort in the treatment D$_{\text {shock}}$, but not in S$_{\text {shock}}$, which is in line with the presence of a repeated-game effect in the effort provision in dynamic but not in static relationships plagued by random shocks—again, as predicted.

For further evidence on the repeated-game effect, we regress the current wage on past-period output and past-period adjustment—see columns (3) and (4) of Table 7. In line with the presence of a repeated-game effect in the wage determination, we find a strong positive correlation between current-period wage and past-period output in the treatment D$_{\text {shock}}$, while there is no significant evidence for such relation in S$_{\text {shock}}$.

We summarize our findings regarding the noise-canceling and the repeated-game effect in the shock treatments as follows::

#### Result 4

In the shock treatments, we find direct evidence for the active part of the noise-canceling effect in dynamic but not in static interactions, and we find direct evidence for the repeated-game effect, both in the wage determination and in the effort provision, in dynamic but not in static interactions.

Result 4 suggests the following explanation for our main result that a dynamic setup eliminates the negative impact of shocks on efficiency: In the static setup, principals offer lower wage payments in the shock than in the no-shock treatment as they anticipate that agents will exert less effort in the presence of shocks. The reason for the lower effort motivation of the agents in the presence of shocks is that agents know that the principals’ reaction in the third stage will be less predictable in the presence of shocks as principals cannot disentangle the agent’s effort from good or bad luck. In the dynamic relationship, there are two counteracting forces: First, part of the noise is canceled out—the noise-canceling effect. And second, current misbehavior (by the agent) can not only be punished by a lower adjustment in the current period but also by a lower wage in the next period—the repeated-game effect. These two effects together seem to restore gift exchange in dynamic relationships plagued by random shocks, but they are unavailable in static relationships.

Summarizing the results of this section, we conclude that there is direct evidence for the active part of the noise-canceling effect (the insurance effect) and for the repeated-game effect in dynamic interactions plagued by random shocks, but not in their static counterpart and that shocks have a less pronounced negative effect on the principal’s wage and on the agent’s effort provision in dynamic than in static interactions. Since the repeated-game effect is also present (and of similar size) in dynamic interactions unaffected by random shocks (see the interaction terms in Tables 19 in online Appendix B), and since it is insufficient to increase efficiency in those interactions, one is tempted to conclude that the noise-canceling effect is the main driver for our result that efficiency is higher in dynamic interactions plagued by random shocks than in their static counterparts but not higher in dynamic interactions not plagued by random shocks than in their static counterparts. In the next section, we address the question whether noise-canceling alone is sufficient to neutralize the negative impact of random shocks.

## The impact of giving regular feedback in form of rewards and punishments

In this section we address the question whether noise-canceling *alone* is enough to neutralize the negative impact of random shocks. To investigate this issue, we run two additional treatments, one with and the other without unobservable random shocks.^9^ In both of these additional treatments the interaction is dynamic—that is, a principal-agent pair remains intact for one block. In contrast to the two D treatments in our main design, in the two additional treatments, principals and agents interact under the *same* contract over the whole block. That is, the first stage is placed at the beginning of each block: the principal offers a wage and states a desired effort, which is valid for each of the five periods in the block. The second stage, in which the agent chooses an effort, and—depending on the treatment—a shock is realized, is played for five periods. In the treatments with a shock, the agent learns the realization of the shock at the end of the respective period. The third stage only takes place at the end of a block. The principal observes the average outcome, and then chooses an adjustment level for all periods in the block.^10^ Again, one period is randomly selected for payment and a new principal-agent pair is formed after the first block.

**Fig. 2:**
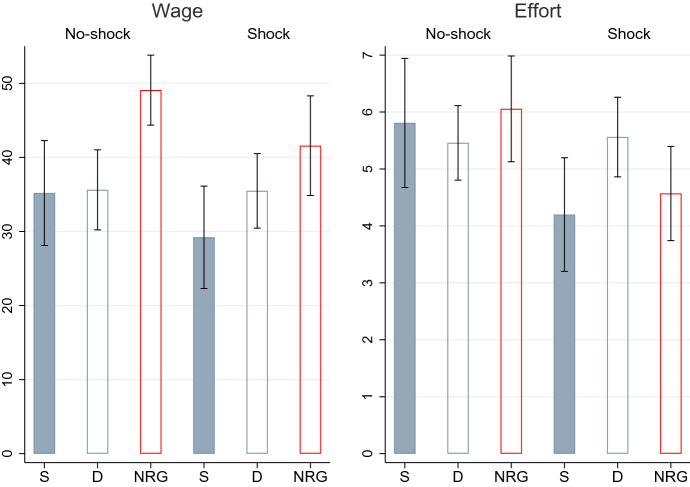
Wage and effort in all treatments, with error bars representing the 95% conf. intervals

In this setup, the two components of the noise-canceling effect—that is, the learning component and the insurance component—potentially are still active, while the repeated-game effect is turned off: the principal does not have an opportunity to reward (punish) the agent for high (low) output in the present period by offering a high (low) wage in the next period and the agent cannot react to a high (low) adjustment in the current period by exerting high (low) effort in the next period. We therefore term the two additional treatments the no-repeated-game-effect (NRG) treatments. The predicted correlation for the NRG$_{\text {shock}}$ treatment is summarized in column (5) of Table 6, and the averages of the main variables are displayed in Table 8 and Fig. 2.

**Table 8:** Averages and Mann-Whitney U-tests regarding decision variables and welfare, NRG treatments

	Wage	Effort	Adjustment	Welfare
*Averages*				
No-shock	49.08	6.06	− 5.69	27.82
	(2.24)	(0.21)	(4.68)	(6.90)
Shock	41.58	4.57	− 1.67	28.93
	(3.18)	(0.39)	(3.13)	(4.65)
*P-values of Mann-Whitney U-tests comparing averages*				
(1) D$_{\text {no-shock}}$ vs. NRG$_{\text {no-shock}}$	0.001	0.26	0.08	0.62
(2) D$_{\text {shock}}$ vs. NRG$_{\text {shock}}$	0.15	0.08	0.01	0.04
(3) NRG$_{\text {no-shock}}$ vs. NRG$_{\text {shock}}$	0.14	0.03	0.57	0.95

Standard errors in parenthesis are based on 18 independent observations; these are also the number of observations used for the Mann-Whitney U-tests

To investigate the impact of removing the repeated-game effect in dynamic gift-exchange relationships without random shocks, we compare the D$_{\text {no-shock}}$ treatment to the NRG$_{\text {no-shock}}$ treatment. Removing the repeated-game effect in the absence of shocks increases av. wage and decreases av. adjustment; however, it does not have a statistically significant effect on av. effort or on av. welfare, see row (1) of the bottom part of Table 8. It seems as if the principals assumed that they need to offer a higher wage to induce high effort, which, however, is not rewarded. In response, the adjustment is reduced.

Next we investigate the impact of removing the repeated-game effect in dynamic gift-exchange relationships with unobservable random shocks by comparing the D$_{\text {shock}}$ treatment to the NRG$_{\text {shock}}$ treatment. Removing the repeated-game effect in the presence of shocks does not decrease the average wage (it even increases the average wage from 35.48 to 41.58; although the increase is large in absolute terms it is not statistically significant—see row (2) of the bottom part of Table 8), while it significantly decreases av. effort, av. adjustment and av. welfare. Here, a similar process as in the no-shock treatments seems to be at work: The principals seem to expect that they need to offer a higher wage to induce high effort, which, however, is not reciprocated. In response, the adjustment is reduced.

To complete the picture, we next analyze within the dynamic relationships whether shocks have the same impact when the repeated-game effect is turned off, by comparing the difference between the D$_{\text {no-shock}}$ and the D$_{\text {shock}}$ treatment to the difference between the NRG$_{\text {no-shock}}$ and the NRG$_{\text {shock}}$ treatment. From the previous section, we know that in the D treatments the presence of unobservable random shocks has neither a significant impact on av. wage, nor on av. effort, nor on av. adjustment. In the NRG setting, the presence of unobservable random shocks decreases the average wage (from 49.08 to 41.58) and increase the average adjustment (from − 5.69 to − 1.67). While the differences loom large in absolute terms they fail to reach statistical significance (see row (3) of the bottom part of Table 8). Average effort is significantly lower in the presence of shocks while average welfare is not significantly affected by them. Turning to the diff-in-diff comparisons, we observe that the impact of shocks on wages is more negative in the NRG treatments, compared to the D treatments, yet not statistically significant (see the differences reported in rows (3) and (5) of Table 5, and the regression results reported in Table 18 in online Appendix B), and the impact of shocks on effort is significantly more negative in the NRG treatments, compared to the D treatments. Av. adjustment is not significantly different between the two settings, as is av. welfare.

The above results suggest that unobservable random shocks have a pronounced negative impact on our main variables of interest in static relationships and in dynamic relationships where the repeated-game effect is turned off. Comparing these two environments we find that the impact of unobservable random shocks on av. wage, effort, adjustment, and welfare is not significantly different between them (see the differences reported in rows (4) and (5) of Table 5, and the regression results reported in Table 20 in online Appendix B). We summarize this evidence to Result 5:

### Result 5

Without shocks, average wage is higher and average adjustment is lower in the no-repeated-game-effect treatment than in the dynamic treatment. Average effort is not significantly different between the treatments, however. In the presence of unobservable random shocks, average wage is again higher in the no-repeated-game-effect than in the dynamic treatment—the difference is not statistically significant, however. Average adjustment and average effort are lower in the no-repeated-game-effect treatment than in the dynamic treatment. In both settings principals seem to assume that they need to offer a higher wage in the no-repeated-game-effect case to induce a high effort. In both cases agents do not react to the higher wage with a higher effort. In response, the average adjustment is reduced compared to the dynamic treatment. Comparing static relationships to dynamic relationships where the repeated-game-effect is turned off we observe that the effect of introducing unobservable random shocks on average effort, average wage and average adjustment is not significantly different between the two settings.

**Table 9:** Panel model of effort, controlling for past-period behavior and shock, only NRG shock treatment

Dep. variable	(1)
	Effort
Wage	0.08$$^{***}$$
	(0.02)
Desired effort	0.13
	(0.10)
Shock$$_{\text {t-1}}$$	− 0.13$$^{**}$$
	(0.06)
Risk aversion	− 0.01
	(0.02)
Inv. period	1.07
	(1.65)
Constant	0.61
	(1.47)
Observations	324

Standard errors in parentheses are clustered on the group level and calculated via bootstrap. Inv. period runs from 1 to 1/10. Risk aversion runs from 1 to 100, with higher numbers indicating less risk aversion. Wage runs from 0 to 100. Effort and desired effort run from 0 to 14. Shock$$_{\text {t-1}}$$ is the shock of the previous period and runs from −2 to 2. The significance levels are: $$^{*}$$ $$p~<~0.10$$, $$^{**}$$ $$p~<~0.05$$, $$^{***}$$ $$p~<~0.01$$

In online Appendix A.3 we investigate the determinants of effort and adjustment in the NRG treatments. For the adjustment stage we regress adjustment on wage, the difference between output and desired effort, the T$_{\text {shock}}$ dummy, interaction terms as well as some control variables. We find that wage has no significant impact on adjustment in either treatment. Output minus desired effort has a positive main effect on adjustment and the effect is significantly smaller in the shock treatment. For the effort stage we regress effort on wage, desired effort, the T$_{\text {shock}}$ dummy, interaction terms of these variables, and control variables. We find that av. effort is lower in the presence of shocks, and there is no significant difference between the treatments in the impact of wage or desired effort on effort. See online Appendix A.3 for more details.

Next we investigate whether there is direct evidence for the active part of the noise-canceling effect (the insurance component), by controlling for past-period shocks in the shock treatments—see Table 9. Indeed, we find evidence for the noise-canceling effect: past-period shock correlates significantly negatively with current-period effort. We record this as Result 6:

### Result 6

In the no-repeated-game-effect treatment we find direct evidence for the active component of the noise-canceling effect (the insurance component) in the effort provision.

Summarizing the results of this section we conclude that there is some direct evidence for the presence of an insurance effect in effort provision in the data of the no-repeated-game-effect treatment plagued by random shocks (Result 6). However, the effect seems to be insufficient to neutralize the negative impact of random shocks on efficiency (Result 5).

Taken together, our results indicate that neither the repeated-game effect alone nor the noise-canceling effect alone is sufficient to alleviate the detrimental effects of unobservable random shocks. What is needed to eliminate the negative effects of shocks is an environment in which both the repeated-game effect and the noise-canceling effect can be active.

## Conclusion

Rubin and Sheremeta (2016) find that reciprocal behavior is heavily depressed if unobservable random shocks blur the relation between effort and output in a static gift-exchange relationship. This result challenges the relevance of gift-exchange for real world employment relationships. In the present paper, we investigated the robustness of this finding by varying the relationship duration from a static interaction between the principal and the agent to a dynamic interaction, and by studying the importance of giving regular feedback in the form of rewards and punishments, in addition with setting the wage payment and having the possibility to observe output regularly.

We have shown that the negative impact of random shocks on wage payment and effort provision is contained if the employment relation is dynamic (the same principal-agent pair interacts over several periods). However, this only holds when the principal has the possibility to give regular feedback in the form of rewards and punishments and by adapting the wage—together with the regular observation of the output. This allows for a repeated-game effect that is important to neutralize the negative impact of unobservable random shocks on reciprocal behavior.

Repeated interaction between the same principal-agent pair, no complete verifiability of the realized effort, but a regular observation of the output, together with the regular opportunity to give feedback by means of paying a bonus or fine, or adapting the wage payment—this is the setting that is most often observed in reality. All in all, our results suggest that reciprocal relationships in these settings are quite robust against the presence of unobservable random shocks.

A possible takeaway from our main finding that a dynamic setup eliminates the negative effect of shocks on gift-exchange is that firms might want to focus on building repeated relationships rather than trying to limit the impact of shocks by investing in greater supervision and more accurate measurement systems.

## Supplementary Information

Below is the link to the electronic supplementary material.

Supplementary file 1 (pdf 985 KB)

## Footnotes

1 This setting reflects the incomplete contracting environment – with its defining feature that the principal cannot enforce the amount of effort the agent exerts when performing the task – much better than the deterministic setting, in which the payment could, in principle, be made contingent on the outcome and thereby on the effort provided.

2 Under the standard assumption of common knowledge that all players are rational and exclusively interested in their material payoffs, the theoretical prediction does not depend on the length of the relationship: The unique subgame-perfect Nash equilibrium (SPNE) of the one-shot game is for the principal to pay no bonus in stage three; for the agent to exert the minimum effort in stage two; and for the principal to pay only the minimum wage in stage one. Since the stage game has a unique SPNE, the unique SPNE of the finitely repeated game is the repetition of the stage game outcome.

3 A consequence of the insurance component is that the agent absorbs part of the shock. If the principal is risk-neutral and the agent is risk-averse (as is typically assumed in the principal-agent literature) then this leads to inefficiencies not considered in the present paper.

4 Indeed, a number of recent studies show that the breach of the implicit agreement in a reciprocal relationship can cause long-lasting adverse effects (see (Heinz et al., 2020; Friebel et al., 2017), among others).

5 The former relationship is predicted because in the repeated relationship the agent might wish to punish (reward) a low (high) adjustment in the present period by exerting low (high) effort in the next period. The latter relationship is predicted because in a dynamic relationship the principal has in fact two punishment mechanisms—current adjustment (which is costly) and future wage (a lower wage actually saves the principal money).

6 For instance, for data-entry and fundraising, individuals can find easier or more difficult items to enter, and they can get assigned a richer or poorer neighborhood to collect donations.

7 Given the difference in the mean effort between the ‘Effort’ and the ‘Effort Shock’ treatment in Rubin and Sheremeta (2016)’s data and given the respective standard deviations, with a sample size of nine observations in each condition and an $\alpha$ of 5%, we have a power of 88% (t-test).

8 Rubin and Sheremeta (2016) find that the presence of shocks decreases av. effort and av. wage while it leaves av. adjustment (statistically) unaffected. Note, however, that the effect size of introducing the shock is very similar to our setting: In Rubin and Sheremeta, the presence of shocks reduces the average wage by 17.72%, the average effort by 26.72%, and the average adjustment by 444.23%. Also, when we compare the shock and the no-shock treatment between the two sets of experiments directly, there is no difference in the wage, effort, or adjustment between the two studies. For the wage level the MWU-test yields $p=0.31$ for the comparison of the no-shock treatments and $p=0.31$ for the comparison of the shock treatments; the respective values for effort and adjustment are $p=0.40$ and $p=0.31$ for the comparison of the no-shock treatments and $p=0.23$ and $p=0.22$ for the comparison of the shock treatments.

9 All treatments were planned ahead, at the same point in time.

10 Importantly, output is not observed by the principal each period; instead, she only observes the average output at the end of a block, before deciding on the adjustment.
