# Supplementary material for: The effect of random shocks on reciprocal behavior in dynamic principal-agent settings
Source: Exp Econ. 2022 Oct 5;26(2):468–88. doi: 10.1007/s10683-022-09771-w (PMC10129933; doi:10.1007/s10683-022-09771-w)
Supplement: Supplementary file 1 — (pdf 985 KB) [file 10683_2022_9771_MOESM1_ESM.pdf]

A Determinants of behavior

A.1 Determinants of adjustment and effort in no-shock treatments

In looking for determinants of behavior in the no-shock treatments, we follow a backward-induction logic – starting with the adjustment stage in which the principal can reward (punish) the agent for high (low) effort by choosing a positive (negative) adjustment. In line with previous literature (Fehr et al., 1997; Rubin and Sheremeta, 2016), we assume that the principal's reciprocity motive is a relative one; i.e., it is a function of the agent's effort, relative to the desired effort. Therefore, we investigate the determinants of the principal's behavior in the third stage by regressing the adjustment on the difference between effort and desired effort (plus some control variables) – see columns (1) and (2) of Table 10. As reported in the main text, the difference between effort and desired effort has a significant positive impact on adjustment in both treatments. Inverse period has a negative effect on adjustment in both environments, however the effect is only significant in the static treatment. That is, controlling for all other reported variables, adjustment is higher in later periods in the static treatment. (It should be noted, however, that the two coefficients do not differ significantly from each other – see the interaction term of 'Inverse period x T_D' in column (5) of Table 10.) A quick glance at the development over time (see figures 3 - 6) suggests that this result is mainly driven by the fact that wage is lower in later periods; also the fitted values in Figure 7 bolster this interpretation. In fact, when including the interaction term 'Wage x inverse period' into Table 10, the significant effect of inverse period on adjustment disappears – see columns (1) and (2) of Table 11 – while none of the other coefficients changes substantially.

In columns (3) and (4) of Table 10, we investigate the determinants of the agent's effort choice in stage 2, by regressing effort on wage and desired effort and on some control variables. As noted in the main text, average effort is significantly higher when the wage is higher, and desired effort has a significant positive impact on effort in both treatments. Inverse period has a significant negative effect on effort in both treatments. That is, controlling for all other reported variables, effort is higher in later periods both in the shock and the no-shock treatment. Again, this effect seems to be driven by the wage – see figures 3 - 6 – and, in fact, the significant effect of inverse period on effort disappears when including the interaction term 'Wage x inverse period' into Table 10 – see columns (3) and (4) of Table 11.

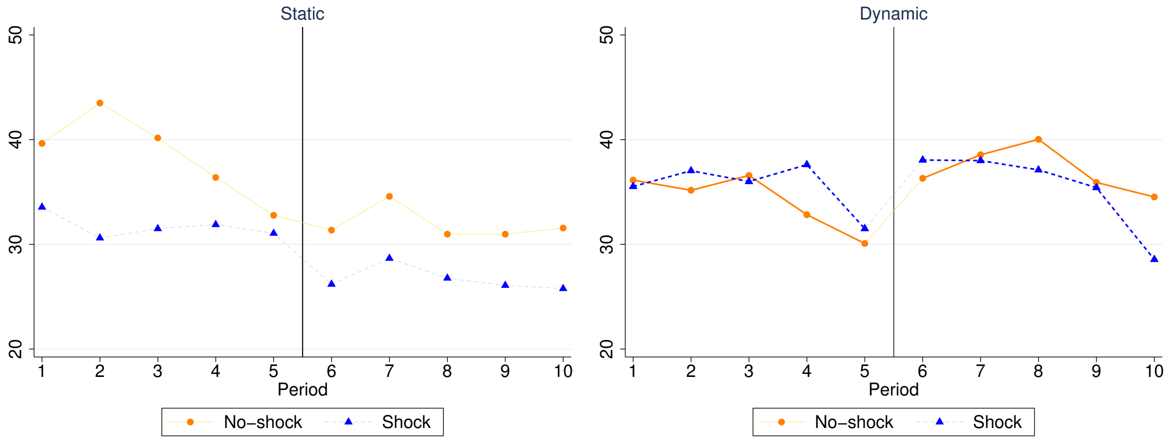

Figure 3: Average wage

Table 10: Panel models of adjustment and effort, no-shock treatments

	(1)	(2)	(3)	(4)	(5)
	S	D	S	D	S and D
Dep. variable:	adjustment	adjustment	effort	effort	adjustment
Wage	0.08	−0.07	0.07***	0.08***	0.06
	(0.09)	(0.11)	(0.01)	(0.01)	(0.11)
Effort - desired effort	2.95***	3.09***			2.83***
	(0.64)	(0.65)			(0.69)
Desired effort			0.11**	0.16***	
			(0.05)	(0.04)	
T_D					3.37
					(6.26)
Wage x T_D					−0.13
					(0.15)
(Effort - desired effort) x T_D					0.30
					(0.94)
Risk aversion x T_D					−0.07
					(0.09)
Inv. period x T_D					8.84
					(9.34)
Risk aversion	0.06	−0.01	0.00	0.00	0.07
	(0.06)	(0.06)	(0.01)	(0.01)	(0.07)
Inv. period	−13.82**	−4.60	−0.80**	−1.17*	−13.40*
	(6.82)	(5.58)	(0.34)	(0.71)	(7.55)
Constant	11.47***	15.22***	2.66***	1.70***	11.88***
	(3.77)	(4.84)	(0.79)	(0.50)	(3.92)
Observations	360	360	360	360	720

Standard errors in parentheses are clustered on the group level and calculated via bootstrap; * $p < 0.10$, ** $p < 0.05$, *** $p < 0.01$. Inv. period runs from 1 to 1/10. Risk aversion runs from 1 to 100, with higher numbers indicating less risk aversion. Wage runs from 0 to 100. Effort and desired effort run from 0 to 14. Adjustment runs from -50 to 50. T_D is a dummy equal to 1 if the treatment is D and zero otherwise.

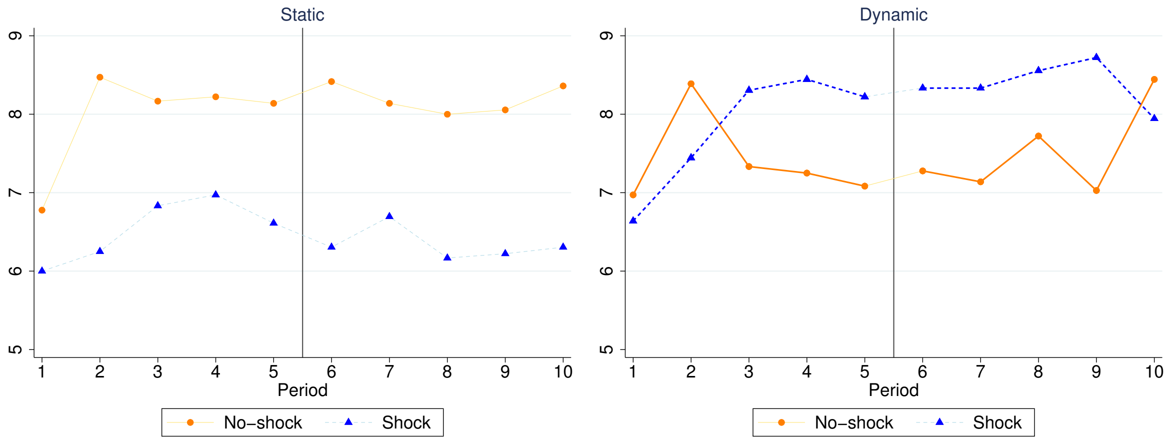

Figure 4: Average desired effort

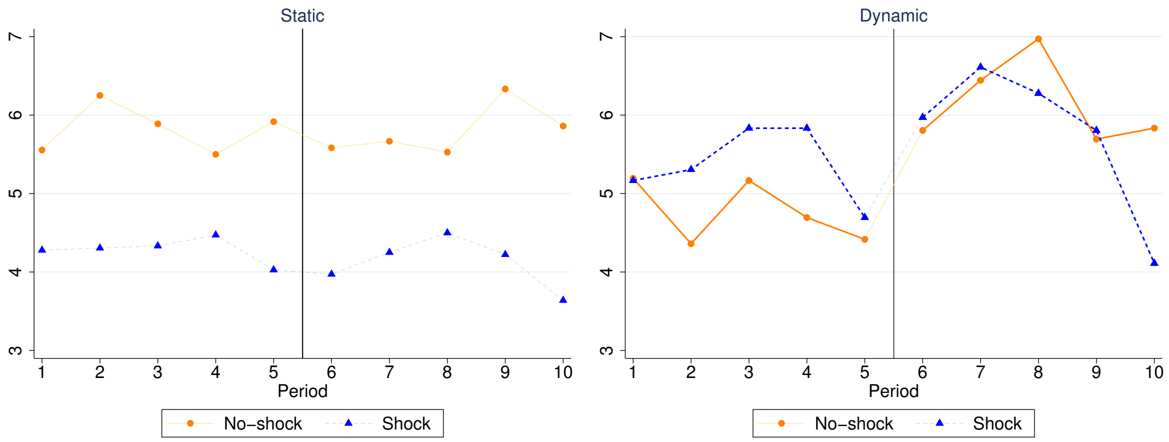

Figure 5: Average effort

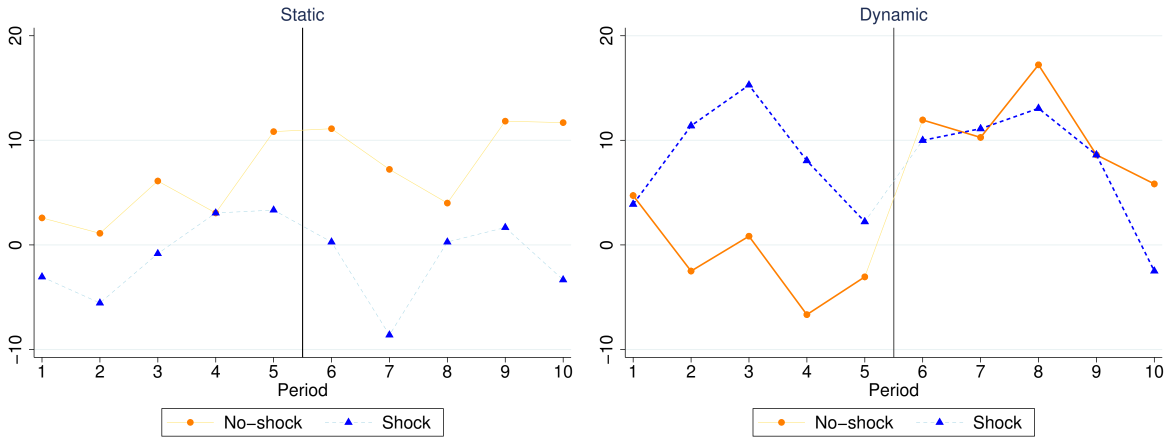

Figure 6: Average adjustment

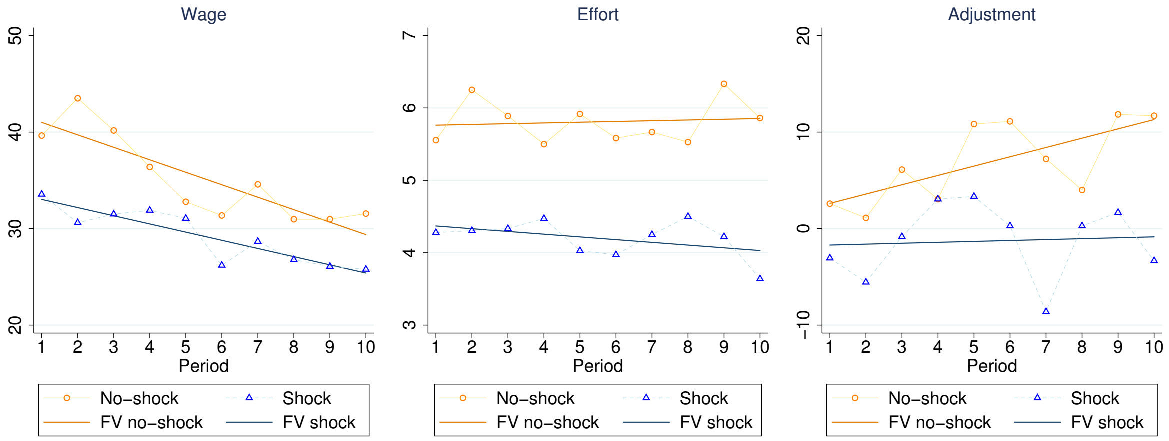

Figure 7: Fitted values for the static treatments, wage, effort, and adjustment

Table 11: Interaction term 'Wage x inv. period' included in Table 10

Dep. variable:	(1)<br>S<br>adjustment	(2)<br>D<br>adjustment	(3)<br>S<br>effort	(4)<br>D<br>effort
Wage	0.18	−0.01	0.08^{***}	0.09^{***}
	(0.14)	(0.16)	(0.01)	(0.02)
Wage x inv. period	−0.29	−0.22	−0.02	−0.02
	(0.32)	(0.31)	(0.02)	(0.04)
Effort - desired effort	2.98^{***}	3.09^{***}		
	(0.65)	(0.65)		
Desired effort			0.11^{**}	0.16^{***}
			(0.05)	(0.04)
Risk aversion	0.06	−0.01	0.00	0.00
	(0.06)	(0.06)	(0.01)	(0.01)
Inv. period	−2.80	3.23	−0.13	−0.56
	(13.66)	(9.57)	(0.87)	(1.40)
Constant	8.34	13.01^{**}	2.46^{***}	1.54^{**}
	(5.67)	(6.23)	(0.87)	(0.69)
Observations	360	360	360	360

Standard errors in parentheses are clustered on the group level and calculated via bootstrap; * $p < 0.10$, ** $p < 0.05$, *** $p < 0.01$. Inv. period runs from 1 to 1/10. Risk aversion runs from 1 to 100, with higher numbers indicating less risk aversion. Wage runs from 0 to 100. Effort and desired effort run from 0 to 14. Adjustment runs from -50 to 50.

A.2 Determinants of adjustment and effort in shock and no-shock treatments

Each column of Table 12 includes data from both the shock and the no-shock treatment. In columns (1) and (2), we regress the adjustment on the difference between output and desired effort, on wage, on a 'T_{shock}' dummy (equal to one if the treatment includes shocks and zero otherwise), on the respective interaction terms, and on control variables. As noted in the main text, there is no significant difference in the impact of 'output - desired effort' between the shock and the no-shock treatments, and as expected, the difference between output and desired effort positively correlates with the adjustment in all treatments – see the separate regressions for the shock and the no-shock treatments – columns (1) and (2) of Table 10 and columns (1) and (2) of Table 13. The interaction term 'Wage x T_{shock}' is negative and (marginally) significant in the static relationship ($p = 0.10$). This means that there is a stronger negative correlation between wage and adjustment in the S_{shock} treatment than in the $S_{no\text{-}shock}$ treatment. However, wage does not have a significant impact on adjustment in either treatment – see the separate regressions for the shock and the no-shock treatments in columns (1) and (2) of Table 10 and columns (1) and (2) of Table 13. Inverse period has a negative effect on adjustment in both sets of treatments, and – as in the no-shock treatments – the effect is only significant for the static interaction. Also, as in the no-shock treatments, including an interaction term between wage and inverse period into the determinants of adjustment shows that the significant effect of inverse period disappears, while the other variables change only marginally – see columns (1) and (2) of Table 14.

In columns (3) and (4) of Table 12 we investigate the determinants of the agent's effort choice, by regressing effort on wage, desired effort, the T_{shock} dummy, interaction terms of these variables, and control variables. As reported in the main text, the impact of wage on effort does not differ significantly between the two treatments, and, as expected, the impact of wage is positive in all settings – see the separate regressions for the shock and the no-shock treatments – columns (3) and (4) of Table 10 and columns (3) and (4) of Table 13. The impact of desired effort on effort does not differ between the shock and the no-shock treatment (the interaction term 'desired effort x T_{shock}' is not statistically significant), and separate regressions for the shock and the no-shock treatments show that desired effort has a significant positive impact only in $S_{no\text{-}shock}$, $D_{no\text{-}shock}$ and D_{shock}, and not in S_{shock} treatment – see columns (3) and (4) of Table 10 and columns (3) and (4) of Table 13. Also here, inverse period has a significant negative effect on effort in both sets of treatments. That is, controlling for all other reported variables, effort is higher in later periods. Again, the effect is mainly driven by the no-shock treatments (compare columns (3) and (4) of Table 10 to columns (3) and (4) of Table 13), and it becomes insignificant when including the interaction term 'Wage x inverse period' – see columns (3) and (4) of Table 14.

A.3 Determinants of adjustment and effort in NRG treatments

Each column in Table 15 includes the shock and the no-shock treatments. In column (1), we regress adjustment on wage, the difference between output and desired effort, the T_{shock} dummy, interaction terms as well as some control variables. 'Output' is av. output from the corresponding block, since that is what the principal observes. As noted in the main text, the impact of wage on adjustment is not significantly different between the two treatments, and it has no significant impact on adjustment in either treatment (see the separate regressions for the no-shock and the shock treatment in columns (1) and (2) of Table 16). Controlling for

Table 12: Panel model of adjustment and effort; shock and no-shock treatments

	(1)	(2)	(3)	(4)
	S	D	S	D
Dep. variable:	adjustment	adjustment	effort	effort
Wage	0.07	−0.07	0.07***	0.08***
	(0.10)	(0.11)	(0.01)	(0.01)
Output - desired effort	2.92***	3.09***		
	(0.66)	(0.68)		
Desired effort			0.11**	0.17***
			(0.05)	(0.04)
T_{shock}	−0.47	−2.72	−0.88	−0.21
	(5.16)	(5.23)	(0.54)	(0.47)
Wage x T_{shock}	−0.23*	0.16	0.01	0.01
	(0.14)	(0.14)	(0.01)	(0.01)
(Output - desired effort) x T_{shock}	0.08	−0.72		
	(0.83)	(0.83)		
Desired effort x T_{shock}			−0.06	−0.01
			(0.07)	(0.06)
Risk aversion	−0.02	−0.01	0.01	0.00
	(0.04)	(0.05)	(0.01)	(0.00)
Inv. period	−8.77**	−5.25	−0.55**	−0.74*
	(4.45)	(4.31)	(27.27)	(0.44)
Constant	14.59***	15.57***	1.98***	1.64***
	(3.99)	(4.42)	(10.20)	(0.44)
Observations	720	720	720	720

Standard errors in parentheses are clustered on the group level and calculated via bootstrap; * $p < 0.10$, ** $p < 0.05$, *** $p < 0.01$. Inv. period runs from 1 to 1/10. Risk aversion runs from 1 to 100, with higher numbers indicating less risk aversion. Wage runs from 0 to 100. Effort and desired effort run from 0 to 14. Adjustment runs from -50 to 50. T_{shock} is a dummy equal to one if the treatment is with shock and zero otherwise. In the no-shock treatments, output corresponds to effort; in the shock treatments, output corresponds to effort plus the shock which runs from -2 to 2.

Table 13: Panel models of adjustment and effort, only shock treatments

Dep. variable:	(1)<br>S<br>adjustment	(2)<br>D<br>adjustment	(3)<br>S<br>effort	(4)<br>D<br>effort
Wage	−0.16	0.09	0.07***	0.09***
	(0.10)	(0.08)	(0.01)	(0.01)
Output - desired effort	3.02***	2.38***		
	(0.54)	(0.48)		
Desired effort			0.05	0.16***
			(0.03)	(0.04)
Risk aversion	−0.10**	−0.01	0.02**	0.00
	(0.05)	(0.07)	(0.01)	(0.01)
Inv. period	−3.89	−5.92	−0.31	−0.30
	(4.52)	(6.23)	(0.41)	(0.48)
Constant	17.18***	13.24***	0.43	1.31**
	(4.09)	(4.52)	(0.67)	(0.61)
Observations	360	360	360	360

Standard errors in parentheses are clustered on the group level and calculated via bootstrap; * $p < 0.10$, ** $p < 0.05$, *** $p < 0.01$. Inv. period runs from 1 to 1/10. Risk aversion runs from 1 to 100, with higher numbers indicating less risk aversion. Wage runs from 0 to 100. Effort and desired effort run from 0 to 14. Adjustment runs from -50 to 50. In the shock treatments, output corresponds to effort plus the shock which runs from -2 to 2.

the different variables, the av. adjustment is significantly lower in the presence of a shock.[11] More importantly, there is a significant difference in the impact of 'Output - desired effort' on adjustment between the two treatments, see the significant coefficient on 'Output - desired effort x T_{shock}' ($p < 0.01$) in Table 15; this is confirmed by separate regressions for the no-shock and the shock treatment – see columns (1) and (2) of Table 16.

In column (2) of Table 15 we regress effort on wage, desired effort, the T_{shock} dummy, interaction terms of these variables, and control variables. As reported in the main text, av. effort is lower in the presence of shocks. There is no significant difference in the impact of wage or desired effort on effort; while wage has a significant positive impact in both treatments (see columns (3) and (4) of Table 16, $NRG_{\text{no-shock}}$, $p < 0.01$; NRG_{shock}, $p < 0.01$), when analyzing the two treatments separately, desired effort shows up weakly significant in the shock treatment, and not significant in the no-shock treatment – see again columns (3) and (4) of Table 16, $NRG_{\text{no-shock}}$, $p = 0.84$; NRG_{shock}, $p = 0.09$.

[11] This is different from what we find in the MWU-test. This is potentially due to the fact that we control for several factors in the regression.

Table 14: Interaction term 'Wage x inv. period' included in Table 12

	(1)	(2)	(3)	(4)
	S	D	S	D
Dep. variable:	adjustment	adjustment	effort	effort
Wage	0.13	0.00	0.08***	0.09***
	(0.12)	(0.14)	(0.01)	(0.01)
Wage x inv. period	−0.17	−0.26	−0.04**	−0.02
	(0.17)	(0.23)	(0.02)	(0.03)
Output - desired effort	2.91***	3.09***		
	(0.66)	(0.68)		
Desired effort			0.11**	0.16***
			(0.05)	(0.04)
T_{shock}	−0.30	−2.45	−0.84	−0.20
	(5.21)	(5.22)	(0.53)	(0.47)
Wage x T_{shock}	−0.24*	0.16	0.01	0.01
	(0.14)	(0.14)	(0.01)	(0.01)
(Output - desired effort) x T_{shock}	0.08	−0.73		
	(0.83)	(0.84)		
Des. eff. x T_{shock}			−0.07	−0.01
			(0.06)	(0.06)
Risk aversion	−0.02	−0.01	0.01	0.00
	(0.04)	(0.05)	(0.01)	(0.00)
Inv. period	−2.74	3.93	0.92	−0.07
	(6.82)	(8.03)	(0.75)	(0.87)
Constant	12.89***	12.81**	1.57**	1.44***
	(4.61)	(5.49)	(0.73)	(0.53)
Observations	720	720	720	720

Standard errors in parentheses are clustered on the group level and calculated via bootstrap; * $p < 0.10$, ** $p < 0.05$, *** $p < 0.01$. Inv. period runs from 1 to 1/10. Risk aversion runs from 1 to 100, with higher numbers indicating less risk aversion. Wage runs from 0 to 100. Effort and desired effort run from 0 to 14. Adjustment runs from -50 to 50. T_{shock} is a dummy equal to one if the treatment is with shock and zero otherwise. In the no-shock treatments, output corresponds to effort; in the shock treatments, output corresponds to effort plus the shock which runs from -2 to 2.

Table 15: Panel model of adjustment and effort, shock and no-shock treatments, NRG treatments

	(1)	(2)
Dep. variable:	adjustment	effort
Wage	-0.10	0.06^{***}
	(0.20)	(0.02)
Output - desired effort	6.07^{***}	
	(0.91)	
Desired effort		-0.01
		(0.12)
T_{shock}	-19.31^{*}	-3.21^{**}
	(11.30)	(1.35)
Wage x T_{shock}	0.23	0.02
	(0.24)	(0.02)
(Output - desired effort) x T_{shock}	-5.53^{***}	
	(1.18)	
Desired effort x T_{shock}		0.16
		(0.14)
Risk aversion	-0.02	0.01
	(0.08)	(0.01)
Inv. period	-65.93^{**}	0.08
	(27.27)	(0.46)
Constant	24.46^{**}	2.76^{**}
	(10.20)	(1.19)
Observations	144	720

Standard errors in parentheses are clustered on the group level and calculated via bootstrap; * $p < 0.10$, ** $p < 0.05$, *** $p < 0.01$. Inv. period runs from 1 to 1/10. Risk aversion runs from 1 to 100, with higher numbers indicating less risk aversion. Wage runs from 0 to 100. Effort and desired effort run from 0 to 14. Adjustment runs from -50 to 50. In the no-shock treatments, output corresponds to effort; in the shock treatments, output corresponds to effort plus the shock which runs from -2 to 2. T_{shock} is a dummy equal to one if the treatment is with shock and zero otherwise. In treatments NRG_{shock} and $NRG_{no\text{-}shock}$, we replace output with average output from the five periods (since that is what the principals observe). Since in these two treatments, the principals only take 2 adjustment decisions (one in period 5, one in period 10) we only have 144 observations in column (1).

Table 16: Panel model of adjustment and effort, separately for the shock and no-shock treatments, NRG treatments

	(1)	(2)	(3)	(4)
Dep. variable:	no-shock adjustment	shock adjustment	no-shock effort	shock effort
Wage	−0.13	0.12	0.06***	0.08***
	(0.20)	(0.16)	(0.02)	(0.02)
Output - desired effort	6.35***	0.60		
	(0.89)	(0.78)		
Desired Effort			−0.02	0.15*
			(0.11)	(0.09)
Risk aversion	−0.16*	0.13	0.03*	−0.01
	(0.09)	(0.12)	(0.02)	(0.02)
Inv. period	−56.50	−76.52*	−0.26	0.42
	(35.05)	(41.48)	(0.45)	(0.79)
Constant	33.47***	0.39	1.73	0.60
	(11.67)	(10.91)	(1.47)	(1.43)
Observations	72	72	360	360

Standard errors in parentheses are clustered on the group level and calculated via bootstrap; * $p < 0.10$, ** $p < 0.05$, *** $p < 0.01$. Inv. period runs from 1 to 1/10. Risk aversion runs from 1 to 100, with higher numbers indicating less risk aversion. Wage runs from 0 to 100. Effort and desired effort run from 0 to 14. Adjustment runs from -50 to 50. In the no-shock treatments, output corresponds to effort; in the shock treatments, output corresponds to effort plus the shock which runs from -2 to 2. In treatments NRG_{shock} and $NRG_{no\text{-}shock}$, we replace output with average output from the five periods (since that is what the principals observe). Since in these two treatments, the principals only take 2 adjustment decisions (one in period 5, one in period 10) we only have 72 observations in column (1) and column (2).

B Additional tables

Table 17: OLS regressions investigating differences between the S and D treatments

	(1) mean wage	(2) mean effort	(3) mean adjustment	(4) mean welfare
T_{shock}	−5.98	−1.61**	−8.23**	−16.16***
	(4.75)	(0.65)	(3.83)	(5.85)
T_D	0.42	−0.35	−2.23	−6.22
	(4.11)	(0.56)	(3.32)	(5.06)
T_{shock} x T_D	5.85	1.71**	11.62**	22.30***
	(5.82)	(0.79)	(4.69)	(7.16)
Constant	35.19***	5.81***	6.96**	39.96***
	(3.36)	(0.46)	(2.71)	(4.13)
Observations	54	54	54	54

Standard errors in parentheses are based on 9 (18) independent observations per treatment, to run an analysis similar to the MWU-tests as for the other results. * $p < 0.10$, ** $p < 0.05$, *** $p < 0.01$. T_{shock} is a dummy equal to one if the treatment is with shock and zero otherwise; T_D is a dummy equal to one if the treatment is dynamic and zero otherwise.

Table 18: OLS regressions investigating differences between the D and NRG treatments

	(1) mean wage	(2) mean effort	(3) mean adjustment	(4) mean welfare
T_{shock}	−7.50**	−1.49***	4.03	1.11
	(3.70)	(0.53)	(4.43)	(6.53)
T_D	−13.47***	−0.60	10.42**	5.93
	(3.70)	(0.53)	(4.43)	(6.53)
T_{shock} x T_D	7.37	1.59**	−0.64	5.04
	(5.23)	(0.74)	(6.26)	(9.24)
Constant	49.08***	6.06***	−5.69*	27.82***
	(2.62)	(0.37)	(3.13)	(4.62)
Observations	72	72	72	72

Standard errors in parentheses are based on 18 independent observations per treatment, to run an analysis similar to the MWU-tests as for the other results. * $p < 0.10$, ** $p < 0.05$, *** $p < 0.01$. T_{shock} is a dummy equal to one if the treatment is with shock and zero otherwise; T_D is a dummy equal to one if the treatment is dynamic and zero otherwise.

Table 19: Difference between D_{shock} and $D_{no\text{-}shock}$, in the effect of $adjustment_{t\text{-}1}$ and $output_{t\text{-}1}$ on wage, and in the effect of $adjustment_{t\text{-}1}$ and $shock_{t\text{-}1}$ on effort

Dep. variable	(1) wage	(2) effort
$Adjustment_{t\text{-}1}$	0.00	0.02***
	(0.05)	(0.01)
T_{shock}	−0.24	−0.22
	(3.78)	(0.29)
$Adjustment_{t\text{-}1}$ x T_{shock}	0.02	0.01
	(0.06)	(0.01)
$Output_{t\text{-}1}$	2.86***	
	(0.36)	
$Output_{t\text{-}1}$ x T_{shock}	−0.27	
	(0.44)	
Wage		0.09***
		(0.00)
Desired effort		0.15***
		(0.03)
$Shock_{t\text{-}1}$		−0.19
		(0.13)
Risk aversion	−0.06*	0.00
	(0.03)	(0.00)
Inv. period	8.34	−2.16**
	(5.59)	(0.95)
Constant	21.63***	1.82***
	(3.22)	(0.42)
Observations	648	648

Notes: Standard errors in parentheses; * $p < 0.10$, ** $p < 0.05$, *** $p < 0.01$. T_{shock} is a dummy equal to one if the treatment is with shock and zero otherwise. Wage runs from 0 to 100. Inv. period runs from 1 to 1/10. Risk aversion runs from 1 to 100, with higher numbers indicating less risk aversion. $Adjustment_{t\text{-}1}$ is the adjustment of the previous period and runs from -50 to 50. $Output_{t\text{-}1}$ is the output of the previous period. In the no-shock treatments, output corresponds to effort; in the shock treatments, output corresponds to effort plus the shock which runs from -2 to 2. Effort and desired effort run from 0 to 14. $Shock_{t\text{-}1}$ is the shock of the previous period.

Table 20: OLS regressions investigating differences between the S and NRG treatments

	(1) mean wage	(2) mean effort	(3) mean adjustment	(4) mean welfare
T_{shock}	-7.50^{**}	-1.49^{***}	4.03	1.11
	(3.64)	(0.55)	(4.87)	(7.30)
T_S	-13.89^{***}	-0.25	12.65^{**}	12.14
	(4.46)	(0.68)	(5.97)	(8.94)
T_{shock} x T_S	1.52	-0.12	-12.26	-17.27
	(6.31)	(0.96)	(8.44)	(12.64)
Constant	49.08^{***}	6.06^{***}	-5.69	27.82^{***}
	(2.57)	(0.39)	(3.45)	(5.16)
Observations	54	54	54	54

Standard errors in parentheses are based on 9 (18) independent observations per treatment, to run an analysis similar to the MWU-tests as for the other results. * $p < 0.10$, ** $p < 0.05$, *** $p < 0.01$. T_{shock} is a dummy equal to one if the treatment is with shock and zero otherwise; T_S is a dummy equal to one if the treatment is static and zero otherwise.

C Instructions

[[[The experiment was run in Austria; the displayed instructions and screenshots are translated from German.]]]

C.1 Title page *[[[for all treatments the same]]]*

Dear participants,

welcome to today's experiment.

Please read the instructions for the experiment carefully. All statements in the instructions are true, and all participants receive exactly the same instructions. Your earnings in the experiment depend on your decisions and potentially the decisions of others. If you have a question, please raise your hand. Your question will then be answered privately. The experiment as well as the data analysis is anonymous.

We ask you not to talk to other participants and to use only the resources and devices that are provided by the conductors of the experiment. Please switch off all electronic devices. In addition, at the computer you are only allowed to use features that are necessary for the experiment. If you do not comply with these rules, you won't be paid in this experiment and you are not allowed to participate in any further experiments.

For today's experiment, funds are provided by the Austrian Science Fund.

The currency used in the experiment is tokens. Tokens will be converted to Euros at a rate of 10 tokens to 1 Euro. You have already received a €9.00 participation fee. Your earnings from the experiment will be incorporated into your participation fee. At the end of today's experiment, you will be paid privately in cash.

The experiment consists of two parts. In total, the two parts will last for around 75 minutes. The two parts of the experiment are completely independent from each other. That is, your payment for part x only depends on decisions that you take in part x, and does not depend on decisions you take in the other part of the experiment.

At the beginning of each part you receive the corresponding instructions. We will read the instructions out loud and will give you time for questions. For a better understanding, in the following we will only use male designations. Those should be understood gender neutral. Thank you a lot for your attention and for participating in today's experiment.

C.2 Treatment S

[[[These are the instructions for the no-shock treatment. When instructions are adapted to the shock treatment, we mark the respective parts with squared brackets.]]]

PART 1

The role assignment

This part consists of 10 periods. In each period you are anonymously assigned to a group, which consists of two participants: participant A and participant B. At the beginning of the first period you will be randomly assigned either as participant A or participant B. You will remain in the same role throughout part 1 of the experiment. So, if you are assigned as participant B in the first period, then you will stay as participant B throughout the 10 periods of part 1.

Independently on your role, at the beginning of each period you are randomly assigned another participant in the other role. That is, if you are participant B, for each period you get assigned another participant A.

Each period will proceed in three stages.

Stage 1

In stage 1, participant A will choose a reward (any integer number between 0 and 100) and a desired effort (any integer number between 0 and 14) for participant B.

An example of the decision screen in stage 1 for participant A is shown below.

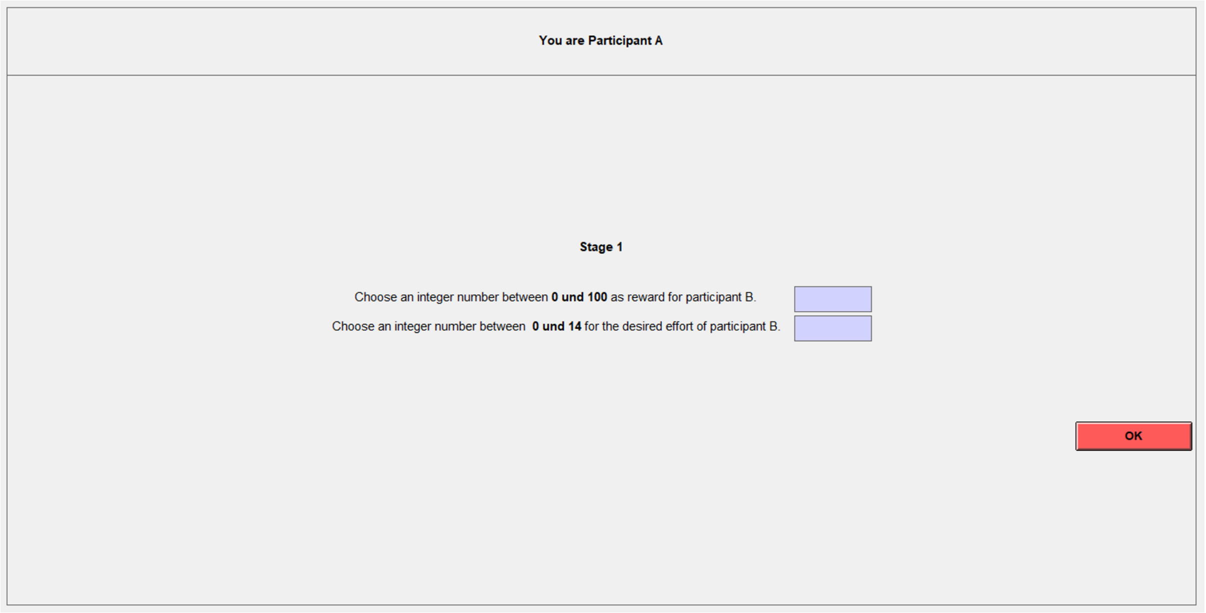

Stage 2

On the screen, participant B is shown the reward and the desired effort chosen by participant A. Then, participant B will choose an effort level (any integer number between 0 and 14).

An example of the stage 2 decision screen for participant B is shown below.

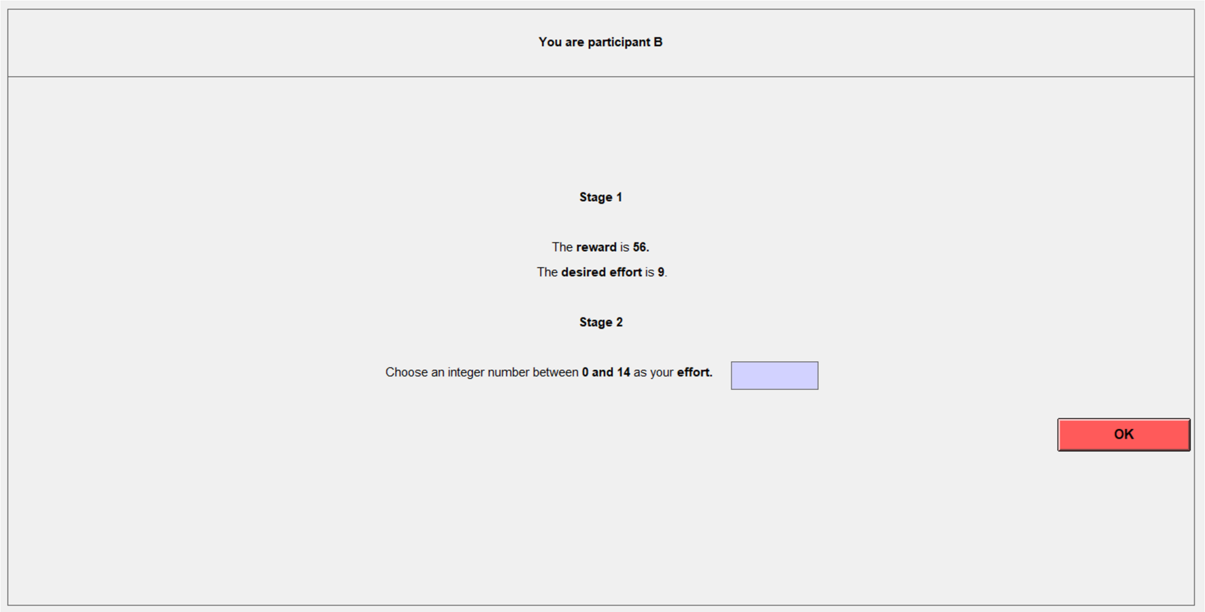

For each effort level chosen by participant B there is an associated cost of effort. The cost of effort can be found in the following table:

Effort	0	1	2	3	4	5	6	7	8	9	10	11	12	13	14
Cost of effort	0	1	2	5	8	13	18	25	32	41	50	61	72	85	98

Note that as effort rises from 0 to 14, costs rise exponentially.

Stage 3

After participant B chooses the effort level, the performance of participant B is determined as follows: Participant B's performance = effort of participant B. Then the computer will display to participant A the performance of participant B on the screen.

[[[Shock treatment: After participant B chooses the effort level, the computer will add to effort a random number to determine the performance of participant B:

Participant B's performance = effort + random number.

The random number chosen by the computer can take a value of -2, -1, 0, 1, or 2. Each number is equally likely to be drawn.

Following the draw of the random number Participants B's performance will be shown to Participant A. Participant A will not know Participant B's actual effort or the random number drawn by the computer.]]]

Then, in the third stage, participant A will choose an adjustment level. The adjustment level must be a multiple of 10, between -50 and 50.

An example of the stage 3 decision screen for participant A is shown on the next picture.

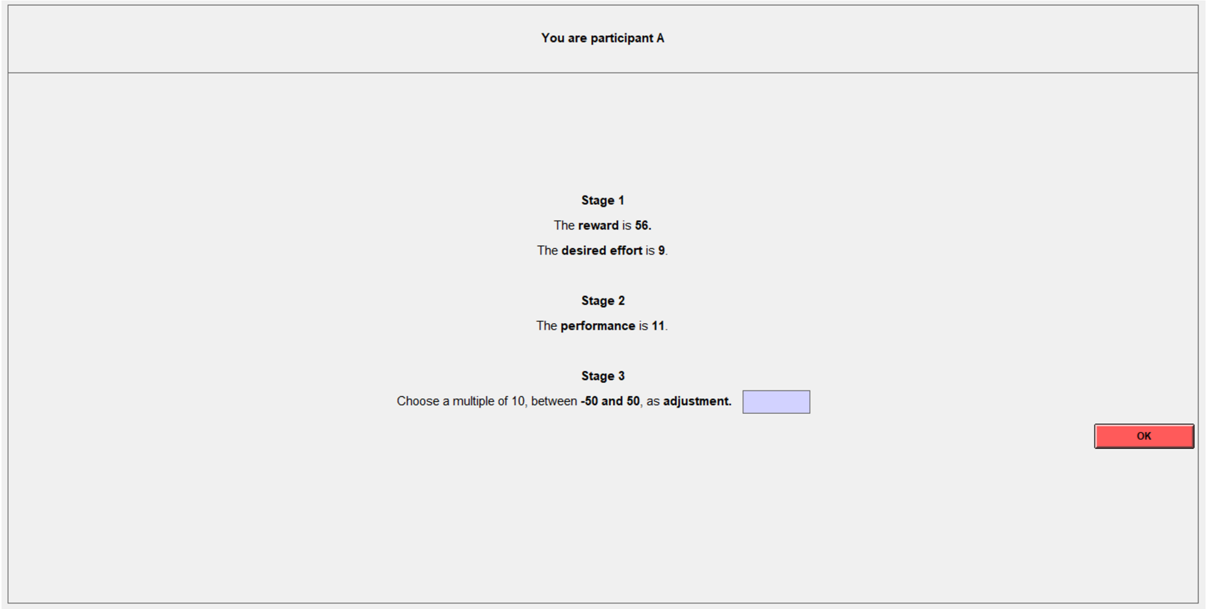

For each adjustment level chosen by participant A there is an associated cost of adjustment. The cost of adjustment can be found in the following table:

Adjustment	-50	-40	-30	-20	-10	0	10	20	30	40	50
Cost of adjustment	5	4	3	2	1	0	1	2	3	4	5

Earnings of participant A

The earnings of participant A depend on the reward chosen by participant A in the first stage, the performance of participant B in the second stage and the adjustment chosen by participant A in the third stage. Specifically, the participant A's earnings are calculated by the following formula:

Participant A's earnings = 10*(performance participant B) – (reward) – (cost of adjustment)
[[[= 10*(effort of part. B+random number) – (reward) – (cost of adj.)]]]

Note that higher participant B's effort implies higher participant B's performance, and thus higher participant A's earnings. On the other hand, a higher reward or a higher cost of adjustment implies lower participant A's earnings.

Earnings of participant B

The earnings of participant B depend on the reward chosen by participant A in the first stage, the cost of the effort chosen by participant B in the second stage and the adjustment chosen by participant A in the third stage. Specifically, participant B's earnings are calculated by the following formula:

Participant B's earnings = (reward) – (cost of effort) + (adjustment)

Note that a higher reward chosen by participant A implies higher participant B's earnings. On the other hand, a higher effort implies higher effort costs and therefore lower participant B's earnings. If participant A chooses a positive adjustment level for participant B then participant B's earnings increase by that adjustment level. However, if participant A chooses a negative adjustment level then participant B's earnings decrease by that adjustment level.

Example 1

Assume the following scenario. In the first stage, participant A chooses a reward of 50 and a desired effort of 7. In the second stage, participant B chooses an effort of 6. [[[Then the computer selects 2 as a random number,]]] so the performance of participant B is 6 [[[8 (6+2)]]]. Then the computer displays to participant A that participant B's performance is 6 [[[8]]]. After observing this information, in the third stage, participant A chooses an adjustment of -40.

Therefore, participant A's earnings = 10*6 – 50 – 4 = 6, since participant B's performance is 6, the reward is 50, and the cost of adjustment of -40 is 4. Finally, participant B's earnings = 50 – 18 – 40 = -8, since the reward is 50, the cost of effort of 6 is 18, and the adjustment is -40.

[[[Therefore, participant A's earnings = 10*8 – 50 – 4 = 26, since participant B's performance is 8, the reward is 50, and the cost of adjustment of -40 is 4. Finally, participant B's earnings = 50 – 18 – 40 = -8, since the reward is 50, the cost of effort of 6 is 18, and the adjustment is -40.]]]

Example 2

Assume the following scenario. In the first stage, participant A chooses a reward of 40 and a desired effort of 6. In the second stage, participant B chooses an effort of 9. [[[Then the computer selects -2 as a random number,]]] so the performance of participant B is 9 [[[7 (9-2)]]]. Then the computer displays to participant A that participant B's performance is 9 [[[7]]]. After observing this information, in the third stage, participant A chooses an adjustment of 30.

Therefore, participant A's earnings = 10*9 – 40 – 3 = 47, since participant B's performance is 9, the reward is 40, and the cost of adjustment of 30 is 3. Finally, participant B's earnings = 40 – 41 + 30 = 29, since the reward is 40, the cost of effort of 9 is 41, and the adjustment is 30.

[[[Therefore, participant A's earnings = 10*7 – 40 – 3 = 27, since participant B's performance is 7, the reward is 40, and the cost of adjustment of 30 is 3. Finally, participant B's earnings = 40 – 41 + 30 = 29, since the reward is 40, the cost of effort of 9 is 41, and the adjustment is 30.]]]

End of the period

At the end of each period, the computer will display to both participants the following information: the reward chosen by participant A, the desired effort chosen by participant A, the performance of participant B, the adjustment chosen by participant A, as well as individual earnings for that period. An example of the outcome screen is shown below.

At the end of each period, the computer will calculate individual earnings.

An example is shown on the following picture.

Once your earnings are displayed on the screen, please record your earnings for the period in your personal record sheet under the appropriate heading.

Important notes

Remember you have already received a €9.00 participation fee. In part 1 of the experiment, depending on a period, you may receive either positive or negative earnings. At the end of part 1 we will randomly select 1 out of 10 periods for actual payment and convert the income thereof to a payment in Euros. If the earnings are negative, we will subtract them from your total earnings. If the earnings are positive, we will add them to your total earnings.

Are there any questions?

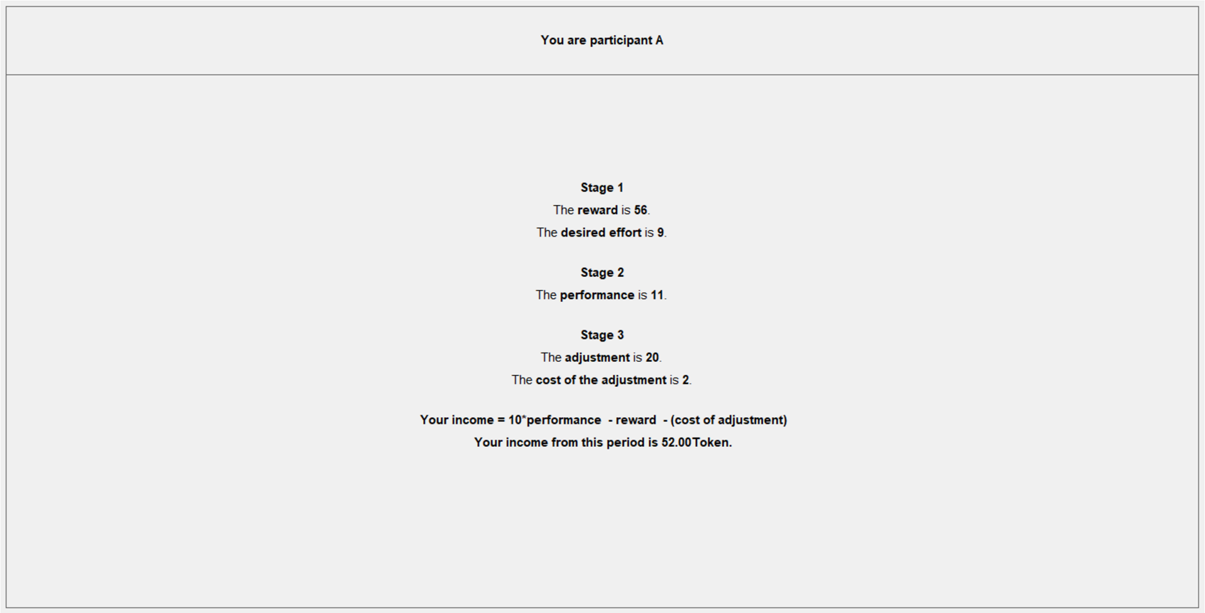

Control questions *[[[implemented in z-Tree]]]*

Question 1: Assume the following scenario. Participant A chooses a reward of 30 and a desired effort of 8. In the second stage, participant B chooses an effort of 7, so the performance of participant B is 7. Then the computer displays to participant A that participant B's performance is 7. After observing this information, in the third stage, participant A chooses an adjustment of 40. What are participant A's earnings? _____(correct: 10*7-30-4 = 36) What are participant B's earnings? _____ (correct: 30-25+40 = 45)

Question 2: Assume the following scenario. Participant A chooses a reward of 40 and a desired effort of 5. In the second stage, participant B chooses an effort of 1, so the performance of participant B is 1. Then the computer displays to participant A that participant B's performance is 1. After observing this information, in the third stage, participant A chooses an adjustment of -50. What are participant A's earnings? _____(correct: 10*1-40-5 = -35) What are participant B's earnings? _____ (correct: 40-1-50 = -11)

[[[Question 1: Assume the following scenario. Participant A chooses a reward of 30 and a desired effort of 8. In the second stage, participant B chooses an effort of 7. Then the computer selects 1 as a random number, so the performance of participant B is 8 (7 +1). Then the computer displays to participant A that participant B's performance is 8. After observing this information, in the third stage, participant A chooses an adjustment of 40. What are participant A's earnings? _____(correct: 10*8-30-4 = 46) What are participant B's earnings? _____ (correct: 30-25+40 = 45)

Question 2: Assume the following scenario. Participant A chooses a reward of 40 and a desired effort of 5. In the second stage, participant B chooses an effort of 1. Then the computer selects -1 as a random number, so the performance of participant B is 0 (1 -1). Then the computer displays to participant A that participant B's performance is 0. After observing this information, in the third stage, participant A chooses an adjustment of -50. What are participant A's earnings? _____(correct: 10*0-40 -5 = -45) What are participant B's earnings? _____ (correct: 40-1-50 = -11)]]]

PART 2

On your computer screen you will see a square composed of 100 numbered boxes, like shown below.

1	2	3	4	5	6	7	8	9	10
11	12	13	14	15	16	17	18	19	20
21	22	23	24	25	26	27	28	29	30
31	32	33	34	35	36	37	38	39	40
41	42	43	44	45	46	47	48	49	50
51	52	53	54	55	56	57	58	59	60
61	62	63	64	65	66	67	68	69	70
71	72	73	74	75	76	77	78	79	80
81	82	83	84	85	86	87	88	89	90
91	92	93	94	95	96	97	98	99	100

Behind one of these boxes hides a mine; all the other 99 boxes are free from mines. You do not know where this mine lies. You only know that the mine can be in any place with equal probability.

Your task is to decide how many boxes to collect. Boxes will be collected in numerical order. So you will be asked to choose a number between 1 and 100.

At the end of the experiment we will randomly determine the number of the box containing the mine. If you happen to have harvested the box where the mine is located – i.e. if your chosen number is greater than or equal to the drawn number – you will earn zero. If the mine is located in a box that you did not harvest – i.e. if your chosen number is smaller than the drawn number – you will earn in euro an amount equivalent to the number you have chosen.

[[[Extra sheet]]]

Effort	0	1	2	3	4	5	6	7	8	9	10	11	12	13	14
Cost of effort	0	1	2	5	8	13	18	25	32	41	50	61	72	85	98

Adjustment	-50	-40	-30	-20	-10	0	10	20	30	40	50
Cost of adjustment	5	4	3	2	1	0	1	2	3	4	5

Participant B's performance = effort [[[+ random number]]]

Participant A's earnings = 10*(performance participant B) – (reward) – (cost of adjustment) [[[= 10*(effort of part. B + random number) – (reward) – (cost of adj.)]]]

Participant B's earnings = (reward) – (cost of effort) + (adjustment)

C.3 Treatment D

[[[These are the instructions for the no-shock treatment. When instructions are adapted to the shock treatment, this is adapted accordingly, like in the S shock / no-shock treatments.]]]

PART 1

The role assignment

This part consists of 10 periods. In each period you are anonymously assigned to a group, which consists of two participants: participant A and participant B. At the beginning of the first period you will be randomly assigned either as participant A or participant B. You will remain in the same role throughout part 1 of the experiment. So, if you are assigned as participant B in the first period, then you will stay as participant B throughout the 10 periods of part 1.

Independently on your role, at the beginning the first and at the beginning of the second block you are randomly assigned another participant in the other role. For one block (five periods), respectively, you are assigned the same partner. That is, if you are participant B, for the first five periods you get assigned one participant A; for the following five periods you get assigned another participant A.

[[[Stage 1, Stage 2, and Stage 3 is as in the S treatment.]]]

C.4 Treatment NRG

[[[These are the instructions for the no-shock treatment. When instructions are adapted to the shock treatment, this is adapted accordingly, like in the S shock / no-shock treatments.]]]

PART 1

The role assignment

This part consists of 10 periods. In each period you are anonymously assigned to a group, which consists of two participants: participant A and participant B. At the beginning of the first period you will be randomly assigned either as participant A or participant B. You will remain in the same role throughout part 1 of the experiment. So, if you are assigned as participant B in the first period, then you will stay as participant B throughout the 10 periods of part 1.

Independently on your role, at the beginning the first and at the beginning of the second block you are randomly assigned another participant in the other role. **For one block (five periods), respectively, you are assigned the same partner.** That is, if you are participant B, for the first five periods you get assigned one participant A; for the following five periods you get assigned another participant A.

Each period will proceed in three stages.

Stage 1

In stage 1, in periods 1 and 6, participant A will choose a reward (any integer number between 0 and 100) and a desired effort (any integer number between 0 and 14) for participant B. **The values chosen in this stage will apply for the following five periods, respectively!**

An example of the decision screen in stage 1 for participant A is shown below.

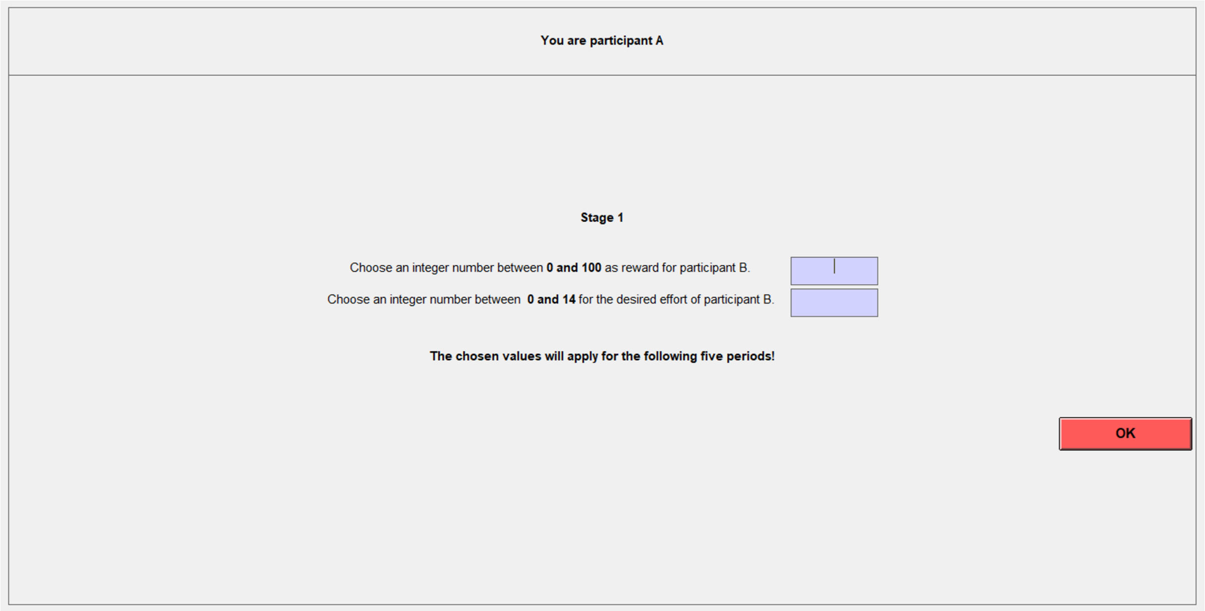

Stage 2

On the screen, participant B is shown the reward and the desired effort chosen by participant A. Then, participant B will choose an effort level (any integer number between 0 and 14). **Participant B chooses an effort level in each of the periods.**

An example of the stage 2 decision screen for participant B is shown below.

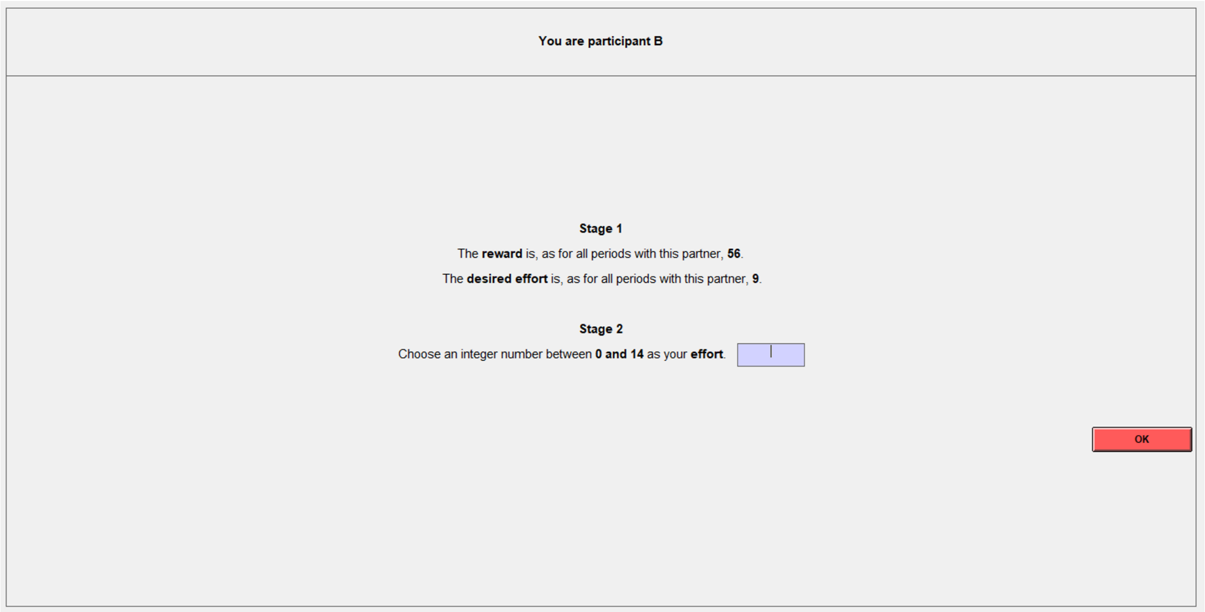

For each effort level chosen by participant B there is an associated cost of effort. The cost of effort can be found in the following table:

Effort	0	1	2	3	4	5	6	7	8	9	10	11	12	13	14
Cost of effort	0	1	2	5	8	13	18	25	32	41	50	61	72	85	98

Note that as effort rises from 0 to 14, costs rise exponentially.

Stage 3

After participant B chooses the effort level, the performance of participant B is determined as follows:

Participant B's performance = effort of participant B.

End of the period

At the end of each period, participant B will be shown the following information on the screen: the reward chosen by participant A, the desired effort chosen by participant A, the performance of participant B, and the earnings for that period – without the "adjustment" (more regarding the adjustment in the next stage). An example for the screen is shown on the following picture.

Once your earnings are displayed on the outcome screen as shown below you should record your earnings for the period on your personal record sheet under the appropriate heading.

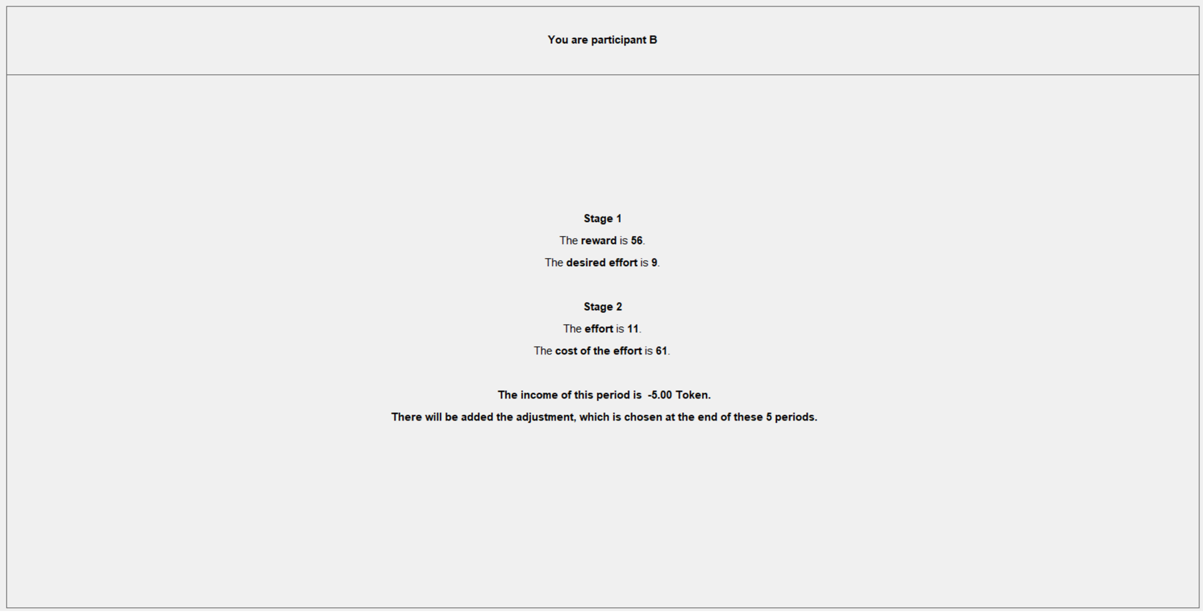

End of the block: the adjustment

At the end of each block – hence, at the end of periods 5 and 10 – the computer shows participant A **the average performance** of the previous five periods. Then participant A can choose an adjustment level. The adjustment level must be a multiple of 10, between -50 and 50.

An example of the decision screen at the end of the block is shown below. **Participant A chooses an adjustment twice: At the end of period 5, and at the end of period 10.**

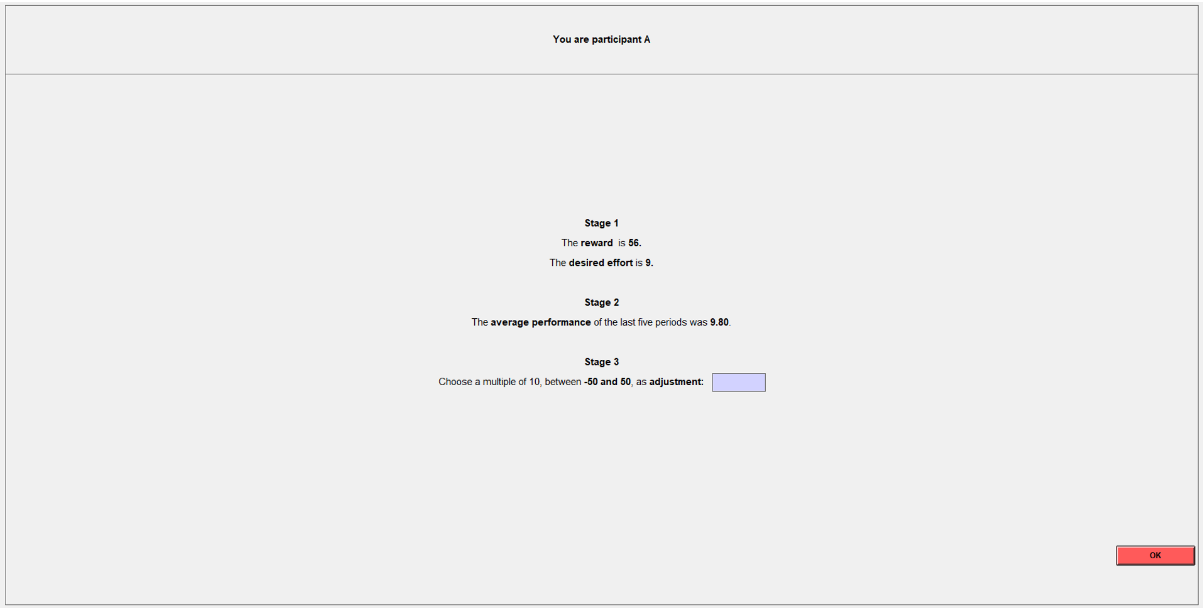

For each adjustment level chosen by participant A there is an associated cost of adjustment. The cost of adjustment can be found in the following table:

Adjustment	-50	-40	-30	-20	-10	0	10	20	30	40	50
Cost of adjustment	5	4	3	2	1	0	1	2	3	4	5

Earnings

At the end of the experiment **one of the 10 periods** is randomly chosen for payment. In this period, the reward of participant A, the effort and the resulting performance of participant B, and the adjustment paid at the end of the respective block as well as the arising costs are used to calculate the incomes. In detail, the incomes are composed as follows:

Earnings of participant A in the respective period

The earnings of participant A depends on three factors: on the reward chosen by participant A, the performance of participant B, and the adjustment chosen by participant A:

Participant A's earnings = 10*(performance participant B) – (reward) – (cost of adjustment)

Higher participant B's effort implies higher participant B's performance, and thus higher participant A's earnings. On the other hand, a higher reward or a higher cost of adjustment implies lower participant A's earnings.

Earnings of participant B in the respective period

The earnings of participant B depend on three factors: the reward chosen by participant A, the effort chosen by participant B; and the adjustment chosen by participant A:

Participant B's earnings = (reward) – (cost of effort) + (adjustment)

A higher reward chosen by participant A implies higher participant B's earnings. On the other hand, a higher effort implies higher effort costs and therefore lower participant B's earnings. If participant A chooses a positive adjustment level for participant B then participant B's earnings increase by that adjustment level. However, if participant A chooses a negative adjustment level then participant B's earnings decrease by that adjustment level.

Example 1

Assume the following scenario. At the beginning of the first block, participant A chooses a reward of 50 and a desired effort of 7.

In period 1, participant B chooses an effort of 5, hence the performance is 5.
In period 2, participant B chooses an effort of 7, hence the performance is 7.
In period 3, participant B chooses an effort of 4, hence the performance is 4.
In period 4, participant B chooses an effort of 6, hence the performance is 6.
In period 5, participant B chooses an effort of 8, hence the performance is 8.

After the first block, hence at the end of period 5, the computer displays to participant A that participant B's average performance is 6. Then, participant A chooses an adjustment of -40. The computer chooses period 4 for payment.

The earnings from participant A in this period is: 10*6 – 50 – 4 = 6: participant B's performance is 6, the reward is 50, and the cost of the adjustment is 4.

The earnings from participant B in this period is: 50 – 18 – 40 = -8: the reward is 50, the cost of effort of 6 is 18, and the adjustment is -40.

Example 2

Assume the following scenario. At the beginning of the second block, participant A chooses a reward of 40 and a desired effort of 6.

In period 6, participant B chooses an effort of 9, hence the performance is 9.
In period 7, participant B chooses an effort of 7, hence the performance is 7.
In period 8, participant B chooses an effort of 9, hence the performance is 9.
In period 9, participant B chooses an effort of 11, hence the performance is 11.
In period 10, participant B chooses an effort of 9, hence the performance is 9.

After the second block, hence at the end of period 10, the computer displays to participant A that participant B's average performance is 9. Then, participant A chooses an adjustment of 30. The computer chooses period 8 for payment.

The earnings from participant A in this period is: 10*9 – 40 – 3 = 47: participant B's performance is 9, the reward is 40, and the cost of the adjustment is 3.

The earnings from participant B in this period is: 40 – 41 + 30 = 29: the reward is 40, the cost of effort of 9 is 41, and the adjustment is 30.

Important notes

Remember you have already received a €9.00 participation fee. In part 1 of the experiment, depending on a period, you may receive either positive or negative earnings. The income from the randomly chosen period will be converted in Euros. If the earnings are negative, we will subtract them from your total earnings. If the earnings are positive, we will add them to your total earnings. Are there any questions?

Control questions *[[[implemented in z-Tree]]]*

Question 1: A) Assume that in period 1 you get assigned the role of participant A. Will your role change in period 2? B) Assume in period 1 you get assigned the role of participant B. For how many periods will you be with the same participant A?

Question 2: Assume the following scenario. Participant A chooses a reward of 30 and a desired effort of 8.
In period 1, participant B chooses an effort of 6. Hence the performance is 6. [[[Then the computer selects 1 as a random number, so the performance of participant B is 7 (6 +1).]]]
In period 2, participant B chooses an effort of 7. Hence the performance is 7. [[[Then the computer selects 0 as a random number, so the performance of participant B is 7 (7 +0).]]]
In period 3, participant B chooses an effort of 9. Hence the performance is 9. [[[Then the computer selects -2 as a random number, so the performance of participant B is 7 (9 -2).]]]
In period 4, participant B chooses an effort of 10. Hence the performance is 10. [[[Then the computer selects 1 as a random number, so the performance of participant B is 11 (10 +1).]]]
In period 5, participant B chooses an effort of 8. Hence the performance is 8. [[[Then the computer selects 2 as a random number, so the performance of participant B is 10 (8 +2).]]]

Then the computer displays to participant A that participant B's average performance is. After observing this information, participant A chooses an adjustment of 20.

Assume period 2 is chosen for payment.

What are participant A's earnings? _____(correct: 10*7-30-2 = 38) What are participant B's earnings? _____ (correct: 30-25+20 = 25)
